# Major vault protein regulates tumor-associated macrophage polarization through interaction with signal transducer and activator of transcription 6

**DOI:** 10.3389/fimmu.2023.1289795

**Published:** 2024-01-09

**Authors:** Chen Yu, Qingmei Zhu, Caijiao Ma, Chuanjin Luo, Longyu Nie, Huanhuan Cai, Qiming Wang, Fubing Wang, Hong Ren, Huan Yan, Ke Xu, Li Zhou, Caiyan Zhang, Guoping Lu, Zhibing Lu, Ying Zhu, Shi Liu

**Affiliations:** ^1^ State Key Laboratory of Virology, Modern Virology Research Center, Frontier Science Center for Immunology and Metabolism, College of Life Sciences, Wuhan University, Wuhan, China; ^2^ Institute of Myocardial Injury and Repair, Wuhan University, Wuhan, China; ^3^ Department of Cardiology, Zhongnan Hospital of Wuhan University, Wuhan, China; ^4^ College of Bioscience and Biotechnology, Hunan Agricultural University, Hunan, Changsha, China; ^5^ Wuhan Research Center for Infectious Diseases and Cancer, Chinese Academy of Medical Sciences, Wuhan, China; ^6^ Shanghai Children’s Medical Center, Affiliated Hospital to Shanghai Jiao Tong University School of Medicine, Shanghai, China

**Keywords:** TAMs, TME, polarization, HCC, MVP, STAT6

## Abstract

Tumor-associated macrophages (TAMs) are critical in the tumor microenvironment (TME) of hepatocellular carcinoma (HCC). Major vault protein (MVP) mediates multidrug resistance, cell growth and development, and viral immunity. However, the relationship between MVP and TAMs polarization has not been clarified in HCC. We found that MVP significantly increased M2-TAMs infiltration levels in tumor tissues of HCC patients. MVP promoted HCC proliferation, metastasis, and invasion by regulating M2 polarization *in vivo* and *in vitro*. Mechanistically, MVP associated with signal transducer and activator of transcription 6 (STAT6) and enhanced STAT6 phosphorylation. STAT6 translocated from the cytosol to the nucleus and regulated M2 macrophage-associated gene transcription. These findings suggest that MVP modulates the macrophage M2 transcriptional program, revealing its potential role in the TAMs of TME.

## Introduction

Hepatocellular carcinoma (HCC) carries high incidence and mortality rates worldwide; the number of HCC patients worldwide rises yearly (1, 2). Insufficient understanding of HCC carcinogenesis may prevent one from making the right treatment choices but immunotherapy sensitivity is more likely to be determined by immune suppression mechanisms operational in the tumor.

Several lines of evidence indicated that cancer immunotherapy based on the tumor immune microenvironment (TME) plays a crucial role in tumorigenesis and progression (3–5). During tumor development, immune cells are recruited into the TME, such as tumor-associated macrophages (TAMs), myeloid-derived suppressor cells (MDSCs) and regulatory T cells (Tregs). The cells with immunosuppressive functions inhibit the proliferation or anti-tumor function of T cells, ultimately contributing to the promotion of tumorigenesis and progression. It has been shown that antibodies targeting VEGFR2 can switch the immunophenotype of TAMs, thereby promoting CD4^+^ and CD8^+^ T-cell function and tumor infiltration (6). In addition to suppressing T cells, immunosuppressive cells infiltrated in TME are directly involved in the process of tumorigenesis, invasion, and metastasis. For example, for many immunotherapies targeting tumor angiogenesis, the number and function of tumor-infiltrating TAMs and MDSCs change as treatment progresses (7). TAMs are commonly infiltrate immune cells that regulate the TME (8). TAMs can be classified as M1-TAMs with anti-tumor functions and M2-TAMs that promote tumors based on functional characteristics. Most tumors possess functional and phenotypic characteristics of M2 macrophages in TME (9). M2-TAMs polarization is a complex dynamic process controlled by T helper 2 cells (Th2) cytokines such as interleukin-4 (IL-4) or interleukin-13 (IL-13) (10). Mechanistically, IL-4 binds to macrophage surface receptors and induces dimerization of the receptor IL-4Rα and γ chains. Cross-phosphorylation occurs at Janus kinase (JAK) 1 and JAK3 coupled to the receptor. The phosphorylated IL-4Rα receptor recruits an intracellular signal transducer and activator of transcription 6 (STAT6) through its SH2 structural domain, and STAT6 recruited to the receptor is phosphorylated by JAK1. Finally, the phosphorylated STAT6 is released into the cytoplasm to form homodimers; it is translocated to the nucleus, where it induces M2 macrophage-related gene transcription, including mannose receptor C type-1 (Mrc1), arginase-1 (Arg1), Retnla (Fizz1), and the chemokine genes 24 (Ccl24) (11). As a result, activated M2-TAMs secrete various cytokines, chemokines, and growth factors to interact with tumor cells or other immune cells, promoting tumor progression (12). Targeting the pro-tumoral M2 macrophages to relieve immune suppression and promote immune-mediated tumor regression is a new direction in HCC therapy.

The vault nanoparticle was discovered in 1986 by researchers using electron microscopy in rat liver coated vesicles (13). Major vault protein (MVP) is the principal constituent; it forms a barrel-shaped structure of the vault with other critical components: small noncoding RNAs (vtRNAs), vault poly (ADP-ribose) polymerase (vPARP), and telomerase-associated protein-1 (TEP1) (14–17). MVP is a drug resistance-associated protein in the lung; studies demonstrated its role in growth, development, and viral immunity (18–20). Our previous work demonstrated that MVP expression was significantly increased in liver cancer tissues compared to normal liver tissues; the high expression of MVP was positively associated with poor outcomes in HCC (21). However, the function of MVP in TAMs of TME-related HCC has not been evaluated.

In this study, we revealed the correlation between MVP and TAMs in TME-related HCC. Further studies demonstrated that MVP is the crucial factor during M2-type macrophages activation. Mechanistically, MVP associates with STAT6, lead to STAT6 phosphorylation and nuclear localization. These findings provide novel research directions for HCC immunotherapy based on M2-TAMs.

## Materials and methods

### Statement of ethics

Clinical sample collection was conducted according to the principles of the Declaration of Helsinki. The Institutional Review Board of Wuhan University approved guidelines for protecting human subjects. All study participants provided written informed consent for collecting samples and subsequent analyses.

Mice were bred and used in specific pathogen-free conditions under protocols approved by Wuhan University. All animal experiments followed the National Institutes of Health Guide for the Care and Use of Laboratory Animals.

### Online database

Tumor Immune Estimation Resource 2.0 (TIMER2.0) (http://timer.cistrome.org/) is a free website based on the original TIMER database. It uses six algorithms to provide immune infiltration level estimation and visualization analysis for TCGA or sequencing data provided by the researcher. The website is divided into three modules: Immune Association, Cancer Exploration, and Immune Estimation. We used the GENE module in Immune Association to analyze the correlation between MVP expression and immune cell infiltration levels of tumors. And we used the GENE_DE module in Cancer Exploration to explore the differential expression between tumor tissues and adjacent nontumorous tissues (ANT) in all TCGA samples.

### Patient samples

All clinical section samples were collected from Zhongnan Hospital of Wuhan University (Wuhan, Hubei, China) and Xiangya Hospital of Zhongnan University (Changsha, Hunan, China), including ten tumor sections and corresponding adjacent nontumorous tissues (ANT) sections of HCC patients. Detailed information on samples is described in **Supplementary Table 2**.

### Plasmids, antibodies, and reagents

Flag-STAT6, Flag-JAK1, Flag-MVP, and Flag-MVP truncated mutants were directly constructed into p3×FLAG-CMV™-14 expression vector by subcloning. HA-MVP, HA-IL-4Rα, HA-STAT6, and HA-STAT6 truncated mutants were subcloned directly into the pKH3-3×HA expression vector. pEGFP-MVP and DsRed-STAT6 were respectively subcloned into pEGFP-C1 and pDsRed-Monomer-N1 vectors. 6×Stat6 DNA-binding motif was synthesized by Kingsley Biotechnology (Nanjing, Jiangsu, China) and cloned into a pGL3-Basic vector. Renilla luciferase reporter vector pRL-TK was purchased from Promega (Madison, WI, USA).

Flow cytometric antibodies for mouse CD45 (103111), F4/80 (123108), CD11b (101227), and CD206 (141705) were purchased from BioLegend. Antibodies for HA (M180-3), Myc (M192-3), and Flag (M185-3L) were purchased from Medical Biological Laboratories. Antibodies for GFP (66002-1-Ig), MVP (16478-1-AP), STAT6 (51073-1-AP), STAT1 (10144-2-AP), Arg-1 (16001-1-AP), CD206 (18704-1-AP), Lamin B1 (66095-1-Ig), GAPDH (60004-1-Ig), beta-actin (66009-1-Ig), ubiquitin (10201-2-AP), and beta-tubulin (66240-1-Ig) were purchased from Protein Tech. The antibody for LRP (1014) (sc23916) was purchased from Santa Cruz Biotechnology. Antibodies for JAK1 (50996) and Phospho-Jak1 (Tyr1034/1035) (3331) were purchased from Cell Signaling Technology. Antibodies for phospho-STAT6 (Tyr641) (CSB-PA000625) and phospho-STAT1 (Tyr641) (CSB-PA050162) were from Cusabio. The antibody for F4/80 (ab300421), Ki67 (ab16667),and CD31 (ab182981) was purchased from Abcam. The antibody for CD68 (BA3638) was purchased from Boster Biological Technology. Antibodies for mouse IgG (Q6004) and rabbit IgG (Q6005) were from DIA-AN Biology. Antibodies for HRP-goat anti-mouse IgG (H+L) (BF03001) and HRP-goat anti-Rabbit IgG (H+L) (BF03008) were purchased from Biodragon. Antibodies for Cy3 AffiniPure Goat Anti-Rabbit IgG (H+L) and Alexa Fluor 488 AffiniPure Goat Anti-Rabbit IgG (H+L) were purchased from Jackson. Antibodies for Dylight 488 Goat Anti-Rabbit IgG (A23220), Dylight 488 Goat Anti-Mouse IgG (A23210), and Dylight 594 Goat Anti-Rabbit IgG (A23420) were purchased from Abbkine. The antibody for HRP rabbit/mouse (K5007) was purchased from DAKO.

Recombinant human IL-4 protein (11846-HNAE), recombinant human IFN-γ protein (11725-HNAS), recombinant mouse IL-4 protein (51084-MNAE), and recombinant mouse IFN-γ protein (50709-MNAH) were purchased from Sino Biological. Lipopolysaccharide (LPS) (L2880) was purchased from Sigma-Aldrich.

### Cell culture and transfection

Human embryonic kidney cells (HEK293T), human myeloid leukemia mononuclear cells (THP-1), human hepatoma cells (Huh-7), mouse hepatocarcinoma cells (Hepa1-6), mouse mononuclear macrophages cells (Raw264.7) and mouse fibroblast cells (L929) were purchased from the China Center Type Culture Collection. HEK293T, Huh-7, Hepa1-6, Raw264.7, and L929 cells were cultured in DMEM (Thermo Fisher Scientific, Waltham, USA) containing 10% fetal bovine serum (FBS) and 1% penicillin-streptomycin (100 U/mL). THP-1 cells were cultured in RPMI1640 (Thermo Fisher Scientific, Waltham, USA) containing 10% FBS and 1% penicillin-streptomycin. All cells were grown in a 37°C cell culture incubator containing 5% CO_2_.

For transfection of HEK293T, the cells were inoculated at 80% in the indicated culture dishes and used polyethylenimine (Polysciences, Philadelphia, USA) according to the manufacturer’s protocol. For macrophage cell lines, Raw264.7 and THP-1 cells were transfected with liposomal transfection reagent (Yeasen, Shanghai, China).

### Murine primary macrophage isolation and polarization

For the isolation of bone Marrow Derived Macrophages (BMDMs) after 6-8 weeks, C57BL/6 mice were euthanized and autopsied. BMDMs were isolated from the mouse tibia and femur using a 1-mL syringe. Subsequently, BMDMs were cultured in DMEM containing 20% L929 supernatant and 10% FBS for 7 days, with cultivation medium changes every 3 days. On the night of the seventh day, BMDMs were incubated in serum-free (SF) medium overnight, after which experiments were performed.

For the isolation of peritoneal macrophages (PMs), sterilized water containing 4% thioglycolate (Sigma-Aldrich, Taufkirchen, Germany) and 0.2% agar were intraperitoneally injected into 6-8 weeks old C57BL/6 mice. After 3 days, the mice were euthanized, and the peritoneal cavity was lavaged with PBS. PMs were incubated in DMEM containing 10% FBS overnight, and the medium was replaced with fresh medium the next day for subsequent experiments.

For M1 macrophage polarization, macrophages were treated with LPS (100 ng/mL) and IFN-γ (50 ng/mL) for 24 hours (h). For M2 macrophages, macrophages were treated with IL-4 (20 ng/mL) for 24 h. For TAMs, macrophages were treated with culture supernatant of Hepa1-6 cells for 24 h.

### Mice and tumor model

Wild-type C57BL/6 (*Wt* mice) was purchased from Center for Disease Control and Prevention (Wuhan, Hubei, China). MVP knockout C57BL/6 (*Mvp^-/-^* mice) was a gift from Professor Erik A.C. Wiemer (Erasmus MC Cancer Institute, University Medical Center Rotterdam) (22). The mice were crossbred with C57BL/6 mice for ten generations to get the heterozygous *Mvp^+/-^* mice in C57BL/6 background. The heterozygous *Mvp^+/-^* C57BL/6 mice performed for self-crossing and screened further to get Homozygous *Mvp^-/-^* C57BL/6 mice. In this study, the groups were composed of mice (*Wt* and *Mvp^-/-^*) from several litters matched for age and sex, and all were derived from background with C57BL/6. All mice were raised in a specific pathogen-free environment at the Center of Animal Experiments at Wuhan University.

For the subcutaneous tumor model, Hepa1-6 cells (3.0 × 10^6^) in 100μl PBS with 30% Matrigel (Corning, Corning, USA) were injected subcutaneously into the right side of 6-8 weeks old male *Wt* or *Mvp^-/-^* mice. For the co-injection assay, BMDMs isolated from mice were polarized into M2 macrophages under IL-4 treatment, and then macrophages (1.5 × 10^6^) in 100μl PBS with 30% Matrigel were co-injected subcutaneously into *Wt* mice with Hepa1-6 cells at ratio of 1:1. For macrophage depletion assay, after Hepa1-6 cells were injected subcutaneously into *Wt* or *Mvp^-/-^* mice, clodronate liposomes (200 μL/mouse) were intraperitoneally injected into mice every 3 days to deplete macrophages. Tumor volume was recorded every two days as follows: V (mm^3^) = (tumor width)^2^ × (tumor length) × π/6. On day 21 post-injection, mice were sacrificed, and tumors were harvested.

### Cell viability, colony formation, wound healing, and transwell assays

For the cell viability assay, Hepa1-6 cells or Huh-7 cells (4.0 × 10^4^/mL) were seeded in 96-well plates (0.1mL/well), and their cell proliferation was measured using a CCK-8 Cell Counting Kit (Vazyme, Nanjing, China) at indicated times.

For the colony formation assay, Hepa1-6 cells or Huh-7 cells (0.5 × 10^3^/mL) were seeded in six-well plates (2mL/well), and fresh medium was changed every three days. On day 21, the supernatant was discarded and washed with PBS. Then, 4% paraformaldehyde was used to fix the cells for 30 min. Finally, cells were stained with 1% crystal violet for 30 min and washed with H_2_O. After drying, the plates were photographed, and the number of cell clones was counted using Image J.

Hepa1-6 cells or Huh-7 cells (1 × 10^6^/mL) were seeded in six-well plates (2mL/well) for the wound healing assay. After the cells were spread over each well, wounds were created with a 1-mL sterile lance tip. Finally, the images were captured with an inverted microscope (Leica, Wetzlar, Germany) at indicated times, and the scratch area was calculated using Image J. The migration rate = (0 h scratch area – scratch area after incubation)/0 h scratch area × 100%.

For the transwell assay, Hepa1-6 cells or Huh-7 cells (5 × 10^5^/mL) were seeded in the upper chamber (0.2mL/well) with Matrigel coating, and the bottom chamber contained DMEM with 20% FBS. After incubating for the indicated times, total cells were fixed with 4% paraformaldehyde and stained with 0.1% crystal violet. Finally, non-invading cells were discarded with cotton swabs before the images of invading cells were captured under an inverted microscope (Leica, Wetzlar, Germany), and the number of invading cells were counted using Image J.

All cells in these assays were maintained in the control medium, *Wt*-M2 macrophages conditioned medium (*Wt* CM), or *Mvp^-/–^* M2/MVP-KD-M2 macrophages conditioned medium (*Mvp^-/-^* CM/MVP-KD CM).

### Flow cytometry

Polarized BMDMs or PMs were incubated in cold PBS containing 2 mM EDTA for 30 min, then the adherent cells were gently scraped off with a spatula and transferred to a centrifuge tube. After centrifugation at 500 g at 4°C, cells were blocked by the commercial Fc-receptors (anti-mouse CD16/32) (Biolegend, San Diego, USA) for 30 min at 4°C. Cell surface proteins were stained with indicated antibodies for 30 min, then, after fixation and lysis using eBioscience™ Fixation/Permeabilization Concentrate (Thermo Fisher Scientific, Waltham, USA) for 30 min; intracellular proteins were stained for 30 min.

For mouse tumor tissues, they were cut into pieces and digested with collagenase IV (2 mg/mL, Sigma-Aldrich, Taufkirchen, Germany) and DNAase I (20 U/mL, Sigma-Aldrich, Taufkirchen, Germany) for 30 min in a 37°C incubator and filtered through a 70 μm cell strainer to obtain a single cell suspension. After lysing the red blood cells using RBC lysis buffer, the obtained cells perform blocking and staining as described above.

All cells samples were resuspended in PBS and analyzed using a flow cytometer (Leica, Wetzlar, Germany) and Flow Jo (v10.6.2).

### Western blot and co-immunoprecipitation analysis

Cells were collected and lysed with RIPA buffer containing 150 mM NaCl, 20 mM Tris-HCl (pH 7.4), 1% NonidetP-40, 0.5% sodium deoxycholate, 1% protease inhibitor (HY-K0010, MCE, Monmouth Junction, USA), and 1% phosphatase inhibitor (K1015, APExBIO, Houston, USA) mixture. The RIPA buffer used in this study does not contain SDS. Lysates were prepared, and protein concentration was measured using a Bio-Rad Protein Assay (Bio-Rad, Hercules, USA). Samples were stored at -20°C until use.

For Co-IP, cells were collected and lysed with NP-40 buffer containing 150 mM NaCl, 20 mM Tris-HCl (pH 7.4), 1% NP-40, and 1% protease and phosphatase inhibitor. Then, 20μl protein A/G agarose was added to the lysates to perform prewash for 2 h, and the appropriate amount of IgG or indicated antibodies was added to the supernatant at 4°C overnight according to the instructions. The following day, the target protein in the lysates was captured by adding protein A/G agarose. The final product was washed five times with NP-40 buffer containing 1M NaCl and stored at -20°C until use. All total protein and Co-IP samples underwent sodium dodecyl sulfate-polyacrylamide electrophoresis, transfer to nitrocellulose membranes, and detection with the indicated antibodies.

### Nuclear extraction

A part of the harvested total cells performed western blot analysis described above and served as whole-cell lysates (WCL). The remaining cells were resuspended with ice-cold buffer A consisting of 10 mM NaCl, 5 mM MgCl_2_, 10 mM Tris-HCl (pH 7.4), 1 mM DTT, 10% protease, and phosphatase inhibitor at 4°C for 20 min, and then 1/20 volume of 10% NP-40 was added and incubated at 4°C for 3min. After centrifugation at 12000 rpm for 5 min at 4°C, the supernatant as cytoplasm extracts and pellet as nuclear pre-extracts were collected. Cytoplasm extracts were stored at -20°C, while nuclear pre-extracts continued with subsequent steps. Before nuclear pre-extracts were resuspended with fresh buffer B consisting of 0.5 mM NaCl, 1.5 mM MgCl_2_, 20 mM HEPES-KOH (pH 7.9), 1 mM DTT, 0.5 mM EDTA, 1% NP-40, 10% protease, and phosphatase inhibitor, they were washed twice with buffer A and once with PBS in order to remove the effects of cytoplasm protein. Then the suspension was vortexed for 15 s and allowed to rest on ice for 30 min. After repeating this step once, they were centrifuged for 30 min at 4°C. Eventually, the supernatant after centrifugation were nuclear extracts, and then maintained at -20°C until use.

### Luciferase assays

THP-1 cells or HEK293T cells were seeded into 12-well plates and co-transfected with pRL-TK, and other indicated plasmids using matching transfection reagents described above. Luciferase activity was measured using a dual luciferase reporter gene kit (Promega, Madison, USA). A Renilla luciferase reporter vector pRL-TK (Promega, Madison, USA) was used as an internal control.

### Quantitative reverse transcription-polymerase chain reaction

Total RNA was obtained with TRIzol reagent (Invitrogen, Carlsbad, USA) following the manufacturer’s protocol, and cDNA was synthesized using the ABScript III RT Master Mix (ABclonal, Wuhan, China). For qRT-PCR, cDNA was amplified by Universal SYBR Green Fast qPCR Mix (ABclonal, Wuhan, China). Relative mRNA expression or fold change was calculated using the 2-ΔΔCT method using GAPDH expression for normalization purposes. All primers used in qRT-PCR are shown in **Supplementary Table 5**.

### Immunofluorescence and immunohistochemistry

HEK293T cells transfected with fluorescence-labeled expression plasmids or PMs were plated into 14-mm confocal dishes (Biosharp, Hefei, China) for immunofluorescence (IF). At the indicated times, the medium was discarded, and 4% paraformaldehyde was added to fix cells for 15 min, 0.2% Triton X-100 to permeabilize for 10 min at 4°C, and 5% bovine serum albumin to block for 1 h at room temperature. Then, cells were incubated with the indicated primary antibodies at 4°C overnight. The next day, fluorescent dye conjugated secondary antibodies were added to the cells and maintained at 37°C for 1 h, then the nuclei were stained with DAPI. The cells were washed three times with PBS between the steps described above. At last, the stained cells were viewed and imaged with a fluorescence microscope (Leica, Wetzlar, Germany).

After performing tissue fixation, embedding, maximal surface sectioning, dewaxing of paraffin sections, and antigen retrieval for tumor tissues of patients and mice, samples were prepared according to the standardized procedures described above. The stained tissue sections were viewed and imaged with a fluorescence microscope (Leica, Wetzlar, Germany).

For immunohistochemistry, the formalin-fixed paraffin sections were dewaxed and rehydrated before being pretreated with 3% H_2_O_2_ for 30 min. Then the antibody binding epitopes of antigens were retrieved by microwave method, and pre-incubated with 3% bovine serum albumin to block non-specific binding. After incubation of the indicated primary and secondary antibodies, 3,3-diaminobenzidine (DAB) was used as a chromogen, and nuclei were counterstained with Harris hematoxylin. The stained tissue sections were viewed and imaged with an inverted microscope (Leica, Wetzlar, Germany).

### Statistics

All images from the inverted and fluorescence microscopes were analyzed using Image J (V2.14.0). All statistical analyses were performed using GraphPad Prism 8 (V8.0.2), all data were expressed as means ± SEM. The specific details of the statistical tests are described in the figure legends. Differences were considered significant when p ≤ 0.05. All experiments represent three repetitions.

## Results

### MVP expression is positively correlated with M2-like TAMs infiltration in HCC

**Figure 1:**
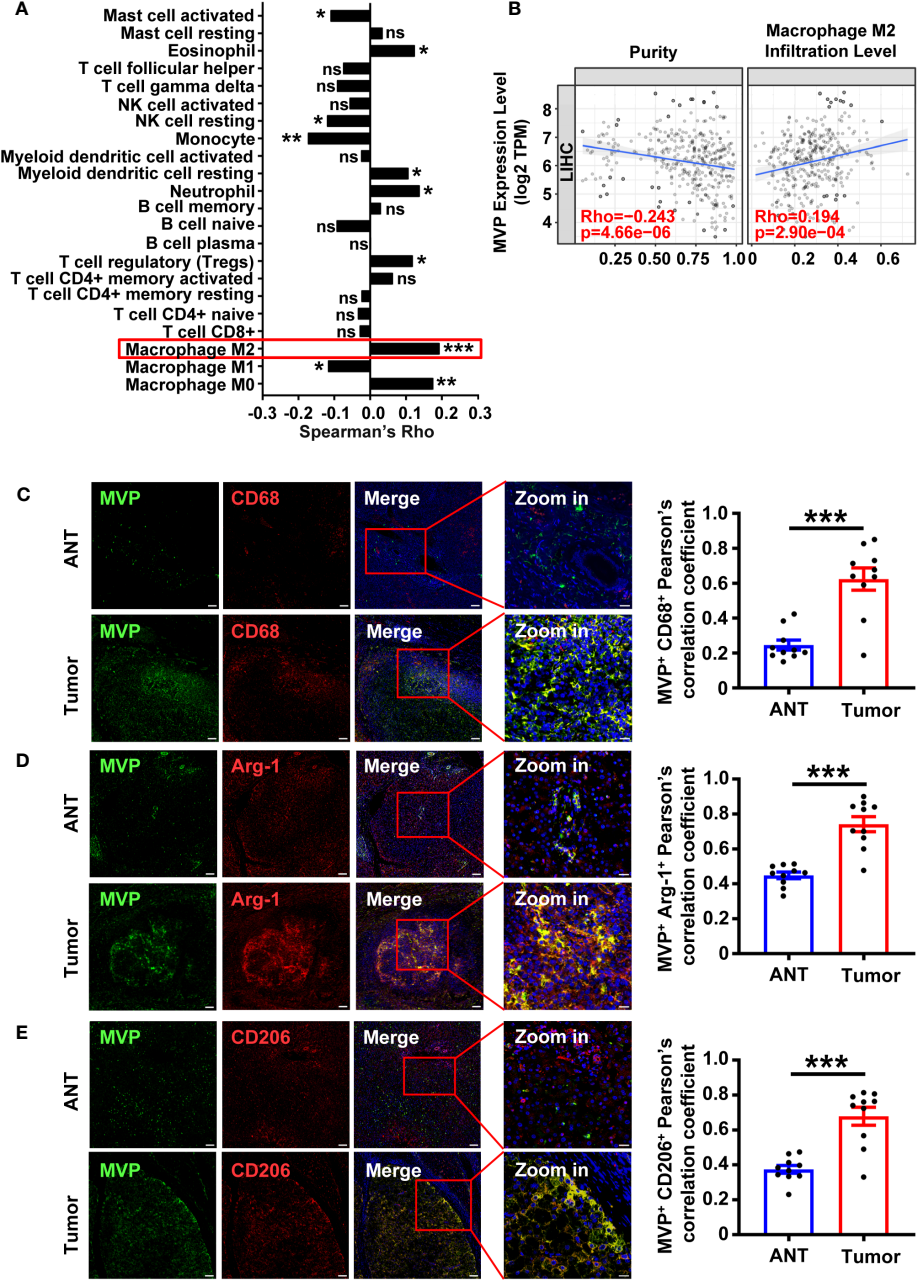
MVP positively correlates with CD68^+^/Arg-1^+^/CD206^+^ TAMs in HCC samples. **(A)** The histogram of Rho between MVP expression and 22 types of immune cell infiltration levels in LIHC from the TIMER2.0 database. The red box highlights the immune cells with the highest positive correlation. **(B)** The scatter plot of the correlation between MVP expression level with tumor purity and M2 macrophage infiltration level determined by the TIMER2.0. TPM: Transcripts Per Kilobase of exon model per Million mapped reads. **(C–E)** Immunofluorescence representative images and co-localization quantitative analysis of MVP (green) with CD68 (red) **(C)**, MVP (green) with Arg-1 (red) **(D)**, and MVP (green) with CD206 (red) **(E)** in adjacent nontumorous tissues (ANT) and HCC tumor tissues (n=10). Scale bar, 100 µm (MVP/CD68/Arg-1/CD206/Merge) and 20 µm (Zoom in). All levels of co-localization are indicated by Pearson’s correlation coefficients calculated using Image J. Data are expressed as means ± SEM, two-tailed Student’s t-test, (***P < 0.001, **P < 0.01, *P < 0.05, ns, no significance). See also **Supplementary Figures 1** and **2**.

To determine the correlation of MVP with the TME of HCC, we calculated the Spearman rank correlation coefficient (Spearman’s Rho) between MVP expression and 22 types of immune cell infiltration using the CIBERSORT algorithm in the TIMER2.0 database (**Figure 1A**; **Supplementary Table 1**). MVP expression positively correlated with M2 macrophages infiltration and had the highest degree among HCC immune infiltrated cells (Rho = 0.194, P < 0.001) (**Figure 1B**). We collected ten paired adjacent nontumorous tissues (ANT) and tumor tissue samples from HCC patients (**Supplementary Table 2**). Immunofluorescence (IF) revealed that MVP expression was more significant in tumor tissues than ANT (**Figures 1C–E**). CD68, CD86, CD206, and arginase 1 (Arg-1) are macrophage markers. As expected, CD68, CD86, CD206, and Arg-1 levels were more significant in tumor tissues than in ANT (**Figures 1C–E**; **Supplementary Figure 1**). Interestingly, there was significant co-localization between MVP and CD68 and Arg-1 and CD206, but not CD86, in HCC patients (**Figures 1C–E**; **Supplementary Figure 1**).

To explore the role of MVP in immune infiltration of M2-like TAMs in other cancers, we examined the MVP expression level and the immune infiltration level of M2-macrophages in The Cancer Genome Atlas (TCGA) tumors from TIMER2.0. The results suggest that MVP expression differed between the tumor and adjacent nontumorous tissues in most human cancers **(Supplementary Figure 2A**; **Supplementary Table 3**). As expected, the expression level of MVP was positively correlated with the infiltration level of M2 macrophages in most types of cancers (**Supplementary Figure 2B**; **Supplementary Table 4**). These results suggest that the MVP expression positively correlated with M2-TAMs infiltration in human tumor samples.

### MVP deficiency inhibits tumorigenicity *in vivo*

**Figure 2:**
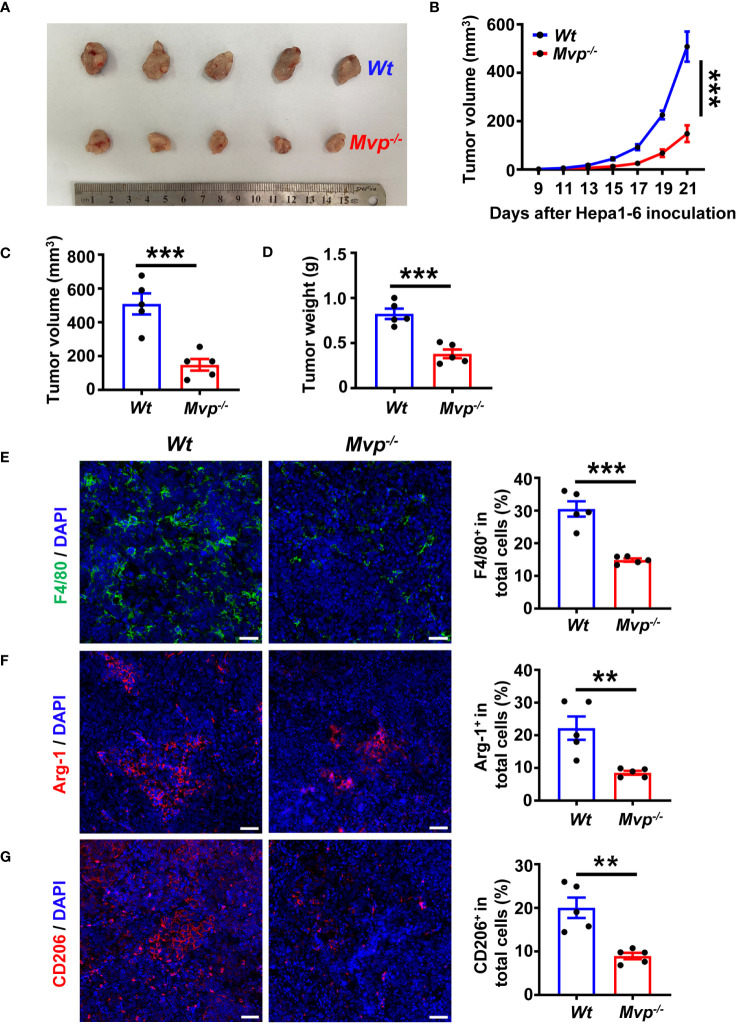
MVP knockout inhibits tumor growth *in vivo*. **(A)** Representative image of the growth of tumors in *Wt* and *Mvp^-/-^* mice (n=5). **(B–D)** 21-day tumor volume change **(B)**, final tumor volume **(C)**, and final tumor weight **(D)** in *Wt* and *Mvp^-/-^* mice (n=5). **(E–G)** Representative IF images and quantitative analysis of F4/80 (green) **(E)**, Arg-1 (red) **(F)**, and CD206 (red) **(G)** in tumor tissues (n=5). Scale bar, 50 µm. The percentage of positive cells out of total cells using Image J. Data in **(B)** are presented as means ± SEM, two-way ANOVA. Data in **(C–G)** are presented as means ± SEM, two-tailed Student’s t-test. (***P < 0.001, **P < 0.01). See also **Supplementary Figure 3**.

To investigate the effect of MVP on tumorigenicity *in vivo*, we employed the MVP knockout (*Mvp^-/-^*) mouse model as described previously (22). In C57BL/6 mice injected with Hepa1-6 cells, the volumes and weights were lower in *Mvp^-/-^* mice compared with wild-type (*Wt*) mice (**Figures 2A–D**). Subsequently, we isolated tumors from mice and performed an IF assay. **Figures 2E–G** shows fewer F4/80, CD206, and Arg-1 positive tumor-infiltrating macrophages were observed in *Mvp^-/-^* mice compared with *Wt* mice. Similarly, Flow cytometry (FC) further revealed that the number of F4/80^+^ CD11b^+^ CD206^+^ macrophages was reduced in tumor tissues of *Mvp^-/-^* mice compared with *Wt* mice (**Supplementary Figures 3A, B**). Thus, these data collectively suggest that MVP deficiency inhibited HCC tumor growth *in vivo*, which may be associated with a reduction in M2-like TAMs infiltration.

### MVP-deficient macrophages inhibit the pro-tumor function of M2 macrophages and the pro-polarization function of Hepa1-6 cells

**Figure 3:**
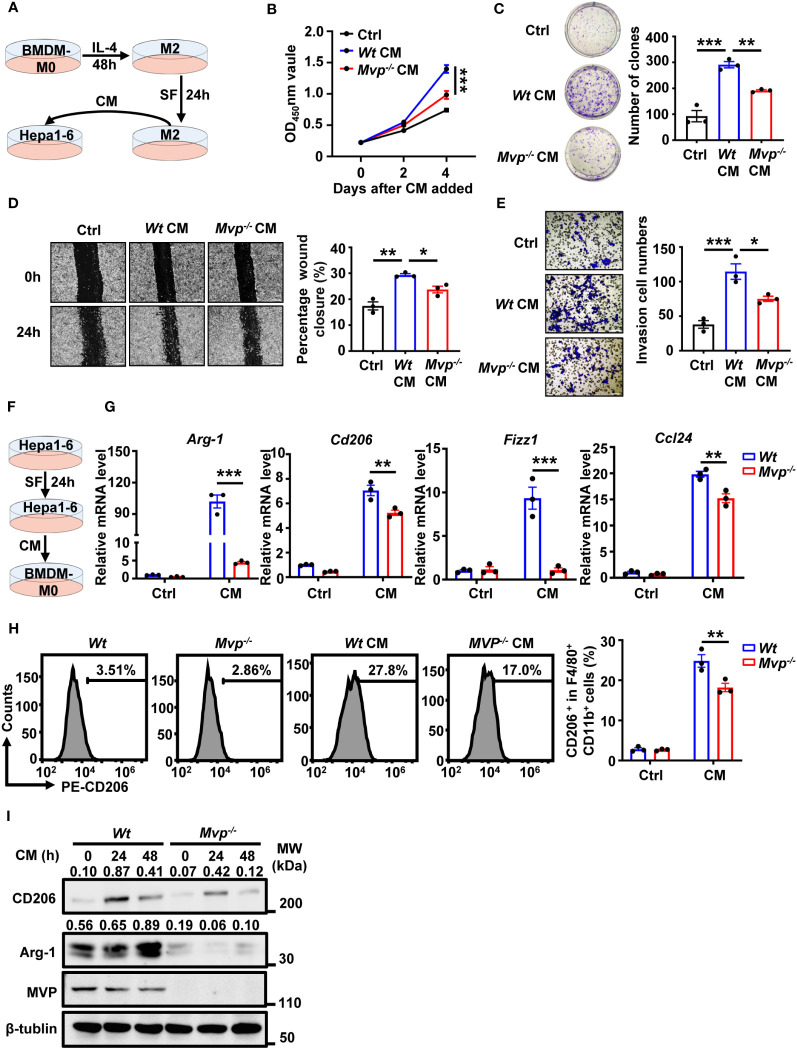
MVP modulates cell growth, motility, and M2-TAMs polarization under CM. **(A)** Model diagram of the macrophage conditioned medium (CM) preparation and treatment. **(B)** Hepa1-6 cells were cultured in CM for 4 days, and cell vitality was measured via CCK-8 assays. **(C)** Representative image and quantitative analysis of clonal formation assay in Hepa1-6 cells cultured in CM for 21 days. The quantitative analysis was performed using Image J. **(D, E)** Hepa1-6 cells were cultured in CM for 24 hours (h) prior to wound healing assay (Scale bar, 50 µm) or transwell invasion assay (Scale bar, 50 µm). The quantitative analysis was performed using Image J. **(F)** Model diagram of the tumor cells CM preparation and treatment. **(G)** qRT-PCR analysis of M2 gene expression in *Wt* and *Mvp^-/-^* macrophages with CM treatment for 24 h. **(H)** FC analysis of CD206 expression in *Wt* and *Mvp^-/-^* macrophages with CM treatment for 24 h. The representative image of the percentage of CD206^+^ cells in F4/80^+^ CD11b^+^ BMDMs (left panel). The percentage of positive cells out of total cells using Flow Jo (right panel). **(I)** Western blot analysis of CD206 and Arg-1 protein expression in *Wt* and *Mvp^-/-^* macrophages with CM treatment at the indicated time. All protein abundance analyses were performed using Image J. Data in **(B–E)** are presented as means ± SEM, n = 3 per condition, one-way ANOVA. Data in **(G, H)** are presented as means ± SEM, n = 3 per condition, two-way ANOVA. (***P < 0.001, **P < 0.01, *P < 0.05). See also **Supplementary Figures 4** and **5**.

We further investigated the effect of *Mvp^-/-^* macrophages on HCC cells *in vitro*. The bone marrow-derived macrophages (BMDMs) of *Wt* and *Mvp^-/-^* mice were stimulated with IL-4 for 48 hours to differentiate into M2 macrophages. Then, M2 macrophages were maintained in a serum-free (SF) medium for 24 hours before collecting culture supernatant (conditioned medium, CM) (**Figure 3A**). There was lower cell viability and colony number in Hepa1-6 cells cultured with CM from *Mvp^-/-^* macrophages than those cultured with CM from *Wt* macrophages (**Figures 3B, C**). Similarly, *Mvp^-/-^* CM inhibited Hepa1-6 cells migration and invasion (**Figures 3D, E**). As expected, we observed a similar phenomenon in Huh-7 cells under THP-1-CM treatment (**Supplementary Figures 4A–E**).

We further investigated the effect of MVP deficiency in macrophages *in vivo* using a co-injection model (**Supplementary Figure 5A**). Compared with *Wt* macrophages, co-injection of *Mvp^-/-^* macrophages had significantly attenuated tumor growth, as well as decreased the expression levels of proliferation marker (Ki67) and blood vessel marker (CD31) (**Supplementary Figures 5B–D**). To further determine whether the pro-tumor effect of MVP requires macrophages *in vivo*, we depleted macrophages by injecting clodronate liposomes (CLOD) (**Supplementary Figure 5E**). The result showed that macrophage depletion eliminated the difference in tumor growth, the expression levels of Ki67 and CD31 between *Wt* and *Mvp^-/-^* mice (**Supplementary Figures 5F–H**).

Crosstalk between tumor cells and immune cells is reciprocal, and some secretions from HCC cells usually cause TAMs to polarize toward the M2 phenotype (23). Therefore, to emulate the HCC microenvironment *in vitro*, we collected Hepa1-6 culture supernatant as CM to treat BMDMs (**Figure 3F**). BMDMs polarized toward the M2-phenotype under CM treatment, while expression of M2 markers Arg-1, Cd206, Fizz1, and Ccl24 mRNA levels were dramatically downregulated in BMDMs from *Mvp^-/-^* mice (**Figure 3G**). FC revealed that MVP-deficient macrophages had lower CD206^+^ cells percentage than *Wt* macrophages under the Hepa1-6 CM treatment (**Figure 3H**). CM-regulated Arg-1 and CD206 protein levels were consistently inhibited in *Mvp^-/-^* macrophages (**Figure 3I**). These findings suggest that MVP is the hub in the crosstalk between tumor and immune cells.

### MVP positively regulates IL-4-mediated M2 macrophage polarization

**Figure 4:**
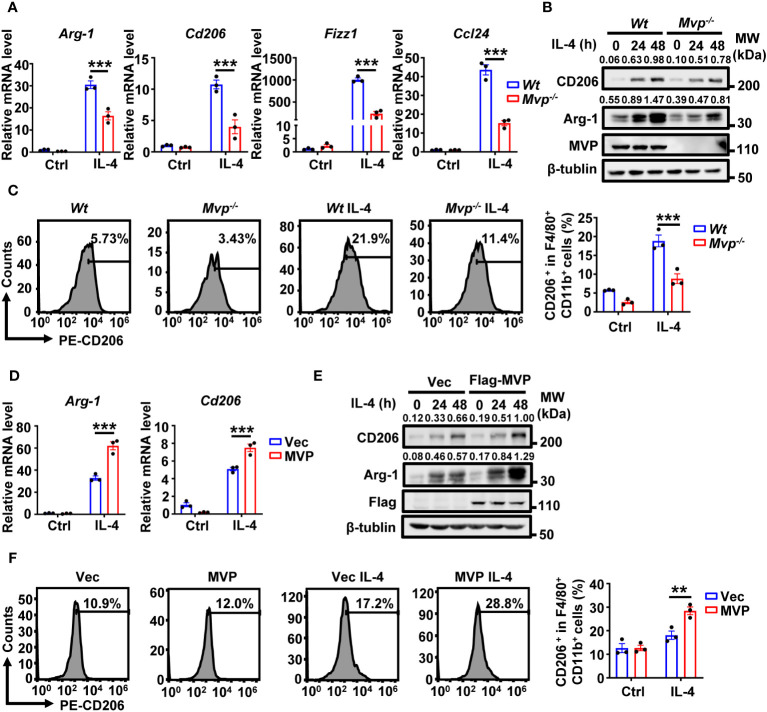
MVP regulates M2 macrophage polarization in response to IL-4 treatment. **(A)** qRT-PCR analysis of M2 gene expression in *Wt* and *Mvp^-/-^* macrophages stimulated with medium or IL-4 for 24 h. **(B)** Western blot analysis of CD206 and Arg-1 protein expression in *Wt* and *Mvp^-/-^* macrophages stimulated with medium or IL-4 at the indicated time. **(C)** FC analysis of CD206 expression in *Wt* and *Mvp^-/-^* macrophages stimulated with medium or IL-4 for 48 h. The representative image of the percentage of CD206^+^ cells in F4/80^+^ CD11b^+^ BMDMs (left panel). The percentage of positive cells out of total cells using Flow Jo (right panel). **(D)** Control vector or pCMV-MVP was transfected into Raw264.7 cells. 24 h after transfection, cells were treated with medium or IL-4 for 24 h and then harvested for qRT-PCR analysis. **(E)** Raw264.7 cells were transfected with indicated plasmids for 36 h. Then, cells were stimulated with or without IL-4 at the indicated time before western blot analysis. **(F)** Raw264.7 cells transfected with indicated plasmids and stimulated with medium or IL-4 for 48 h. The representative image of the percentage of CD206^+^ cells in F4/80^+^ and CD11b^+^ total cells (left panel). The percentage of positive cells out of total cells using Flow Jo (right panel). All protein abundance analyses were performed using Image J. Data in this figure showed the means ± SEM, n = 3 per condition, two-way ANOVA. (***P < 0.01, **P < 0.01). See also **Supplementary Figure 6**.

Lipopolysaccharide (LPS) and interferon-γ (IFN-γ) induce the polarization of classically activated M1 macrophages, while IL-4 induces the polarization of alternatively activated M2 macrophages (24). We investigated the role of MVP in IL-4-regulated M2 macrophage polarization. MVP knockout decreased IL-4-induced M2 macrophage markers, including Arg-1, Cd206, Fizz1, and Ccl24 (**Figures 4A, B**). Similar results were obtained by FC (**Figure 4C**). In contrast, MVP overexpression increased IL-4-induced M2 macrophage markers at the mRNA and protein levels (**Figures 4D, E**). Consistently, MVP overexpression enhanced IL-4-induced CD206^+^ cells percentage in F4/80^+^ CD11b^+^ macrophages. (**Figure 4F**).

To determine whether this phenomenon applies to human-derived macrophages, we constructed MVP knockdown THP-1 cells and induced M2 macrophages using human IL-4. As expected, MVP knockdown downregulated mRNA expression levels of human-derived M2 macrophage marker genes (TGM2, CCL22, and CD163) in THP-1 cells (**Supplementary Figure 6A**). We further investigated the role of MVP in the polarization of M1 macrophages. MVP overexpression inhibited LPS- and IFN-γ**-**induced mRNA expression of M1 macrophage markers (Il-6, Tnf-α, and Cd86) (**Supplementary Figure 6B**). Conversely, MVP knockdown upregulated LPS and IFN-γ**-**induced polarization of M1 macrophages (**Supplementary Figure 6C**). STAT1 is a signaling protein in the classical pathway of IFN-induced M1 polarization. As expected, MVP knockout promoted IFN-γ-mediated STAT1 phosphorylation (**Supplementary Figure 6D**). These findings suggest MVP positively regulates IL-4-induced M2 macrophage polarization but negatively regulates LPS- and IFN-γ-induced M1 macrophage polarization.

### MVP associates with the binary complex JAK1/STAT6

**Figure 5:**
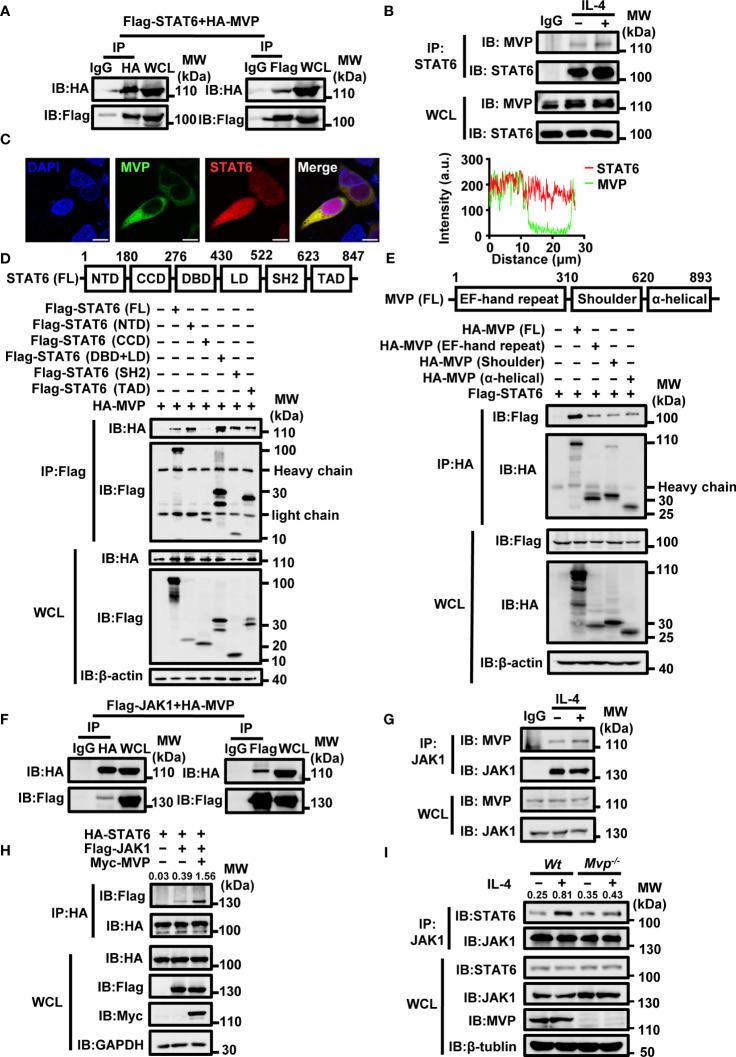
MVP interacts with STAT6 and JAK1. **(A)** HA-tagged MVP and Flag-tagged STAT6 were co-transfected into HEK293T at 36 h post-transfection, and the cells were collected for Co-immunoprecipitation (Co-IP) and immunoblot analyses. **(B)** BMDMs were cultured in SF medium overnight after the formation of adherent cells. The next day, cells were stimulated with medium or IL-4 for 30 min and collected for Co-IP and immunoblot analyses. **(C)** HEK293T cells were transfected with pEGFP-MVP and DsRed-STAT6 for 36 h and then collected for IF assays. Representative image of MVP (green) and STAT6 (red), Scale bar, 10 µm (left panel). The quantitative analysis of co-localization using Image J (right panel). **(D)** Schematic diagram of structural domains and truncated constructs of STAT6 full-length (FL) (upper panel). HEK293T were co-transfected with HA-tagged MVP and truncated mutants of Flag-tagged STAT6 for 36 h. Then cells were collected for Co-IP and immunoblot analyses (lower panel). **(E)** Experiments were performed similarly to those in **(D)**, except the indicated truncated constructs of MVP were used. **(F)** Flag-JAK1 and HA-MVP were co-transfected into HEK293T for 36 h, and then the cells were harvested for Co-IP and immunoblot analyses. **(G)** BMDMs were stimulated with or without IL-4 for 30 min and collected for Co-IP and immunoblot analyses. **(H)** HEK293T cells were transfected with indicated plasmids for 36 h prior to Co-IP and immunoblot analyses. **(I)** *Wt* and *Mvp^-/-^* BMDMs were stimulated with medium or IL-4 for 30 min prior to Co-IP and immunoblot analyses. All protein abundance analyses were performed using Image J. All experiments were repeated at least three times with similar results. See also **Supplementary Figure 7**.

Previous studies indicated that STAT6 is a critical signal regulator in M2 macrophage polarization (25–27). We hypothesized that MVP would directly interact with STAT6. Co-immunoprecipitation (Co-IP) and reverse Co-IP suggest that MVP interacted with STAT6 (**Figure 5A**). Endogenous Co-IP experiments suggest that MVP interacted with STAT6, and this interaction was enhanced in the presence of IL-4 stimulation (**Figure 5B**). IF experiments suggest that MVP co-localized with STAT6 in HEK293T cells (**Figure 5C**). The MVP and STAT6 interaction was not cell-type-specific because similar results were observed in mouse primary macrophages (**Supplementary Figure 7A**). To map the region of STAT6 that interacts with MVP, we constructed truncation mutants of STAT6 (**Figure 5D**, upper panel). Co-IP experiments showed that MVP interacted with all the domains of STAT6 except for the CCD domain (**Figure 5D**, lower panel). To map the region of MVP that interacts with STAT6, we constructed truncation mutants of MVP (**Figure 5E**, upper panel). Co-IP experiments showed that STAT6 interacted with all single domains of MVP (**Figure 5E**, lower panel).

JAK1 and IL-4Rα are structurally and functionally related to STAT6, and the binding of JAK1 with IL-4Rα is essential for IL-4-mediated activation of STAT6 (28). Therefore, we examined MVP’s role in forming IL-4Rα/JAK1/STAT6 complexes. MVP interacted with JAK1 but not IL-4Rα (**Figure 5F**; **Supplementary Figure 7B**). Endogenous Co-IP experiments revealed that MVP interacts with JAK1 in response to IL-4 treatment (**Figure 5G**). Competitive Co-IP experiments demonstrated that MVP enhanced the JAK1 and STAT6 interaction but not the JAK1 and IL-4Rα interaction (**Figure 5H**; **Supplementary Figure 7C**). Similarly, compared with *Wt* mouse primary macrophages, MVP deletion diminished the binding of JAK1 to STAT6 in response to IL-4 treatment (**Figure 5I**). Based on these findings, we propose the following model: in M2 macrophages, MVP interacts with JAK1, recruiting STAT6 to form the ternary complex JAK1/MVP/STAT6.

### MVP regulates IL-4-mediated STAT6 phosphorylation and nuclear localization

**Figure 6:**
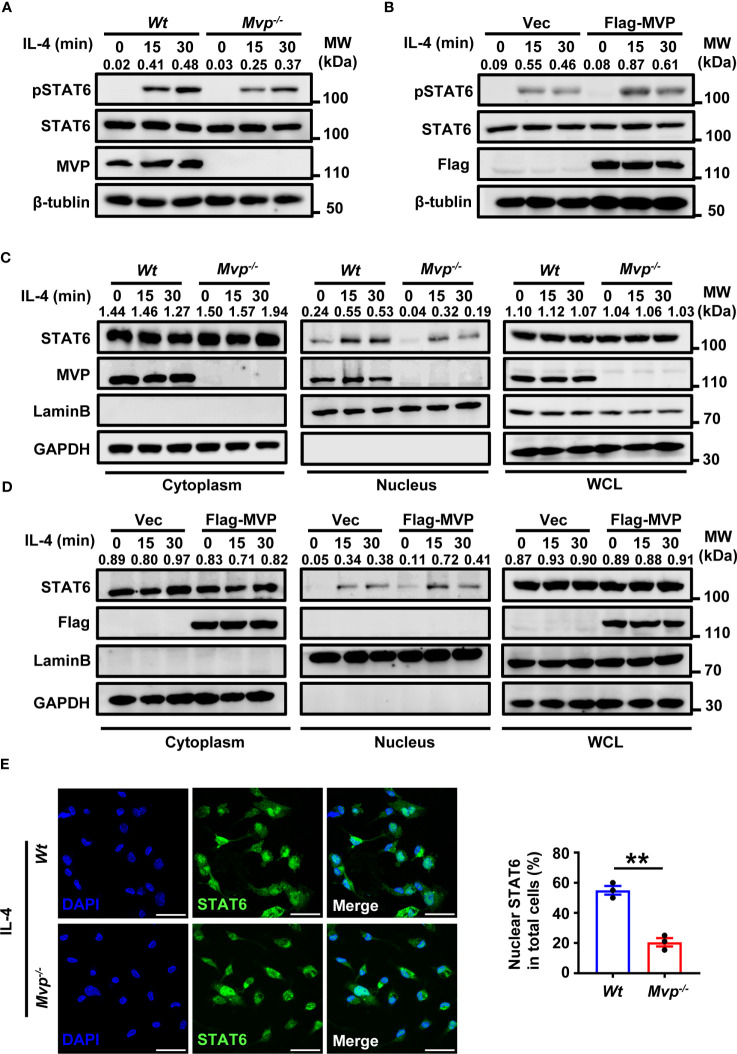
MVP enhances the phosphorylation and nuclear translocation of STAT6. **(A)** *Wt* and *Mvp^-/-^* BMDMs were stimulated with or without IL-4 at the indicated times before western blot analysis. **(B)** Raw264.7 cells were transfected with Flag-tagged MVP or vector for 36 h, then stimulated with phosphate-buffered saline (PBS) or IL-4 for 30 min. Immunoblot analyses were performed with the indicated antibodies. **(C)** *Wt* and *Mvp^-/-^* BMDMs were stimulated with IL-4 for the indicated time. The whole cell lysates (WCL), cytosolic and nuclear extracts were prepared and subjected to western blot analyses. Lamin B and GAPDH were used as nuclear and cytosolic fractions markers, respectively. **(D)** Experiments were performed similar to those in **(C)**, except Raw264.7 cells were transfected with Flag-MVP for 36 h. **(E)** IF of STAT6 in *Wt* and *Mvp^-/-^* PMs stimulated with IL-4. Representative image of STAT6 (green). Scale bar, 20 µm (left panel). The percentage of nuclear STAT6 positive cell numbers was counted (right panel). All protein abundance analyses were performed using Image J. All experiments were repeated at least three times with similar results. Data are expressed as means ± SEM, n = 3, two-tailed Student’s t-test. (**P < 0.01). See also **Supplementary Figure 8**.

Because JAK1 and STAT6 activation is essential for IL-4-mediated polarization of M2 macrophages, we explored the effect of MVP on post-translational modifications of JAK1 and STAT6. STAT6 phosphorylation (but not JAK1 phosphorylation) decreased in *Mvp^-/-^* macrophages under IL-4 treatment (**Figure 6A**; **Supplementary Figure 8A**). Conversely, MVP overexpression enhanced STAT6 phosphorylation in response to IL-4 treatment (**Figure 6B**). Similarly, Hepa1-6 CM promoted STAT6 phosphorylation in *Wt* macrophages but not *Mvp^-/-^* macrophages (**Supplementary Figure 8B**). There was significantly less STAT6 phosphorylation in Hepa1-6 cells induced tumor tissues from *Mvp^-/-^* mice than in *Wt* mice (**Supplementary Figure 8C**). Ubiquitination also controls the STAT6 function (29, 30). However, MVP did not affect STAT6 ubiquitination in response to IL-4 treatment (**Supplementary Figure 8D**). We examined the effect of MVP on the dimerization formation and translocation of STAT6 from the cytosol to the nucleus, which is a hallmark of STAT6 activation. MVP overexpression weakly promoted STAT6 dimerization formation (**Supplementary Figure 8E**). MVP deletion depressed STAT6 nuclear translocation in the presence of IL-4 stimulation, while MVP overexpression promoted the nuclear translocation of STAT6 (**Figures 6C, D**). Similar results were obtained by IF experiments (**Figure 6E**). Similarly, CM from Hepa1-6 cells increased nuclear translocation of STAT6, and deletion of MVP decreased the effectiveness of this process (**Supplementary Figure 8F**). To determine whether MVP affected STAT6 binding activity, we designed a luciferase reporter plasmid containing six consecutive STAT6 preferring DNA-binding motifs (**Supplementary Figure 8G**). Luciferase assays showed MVP knockdown inhibited IL-4-induced STAT6 activity, while MVP overexpression enhanced IL-4-induced STAT6 activity (**Supplementary Figures 8H, I**). These findings suggest that MVP induces the phosphorylation of STAT6, leading to STAT6 nuclear translocation.

## Discussion

**Figure 7:**
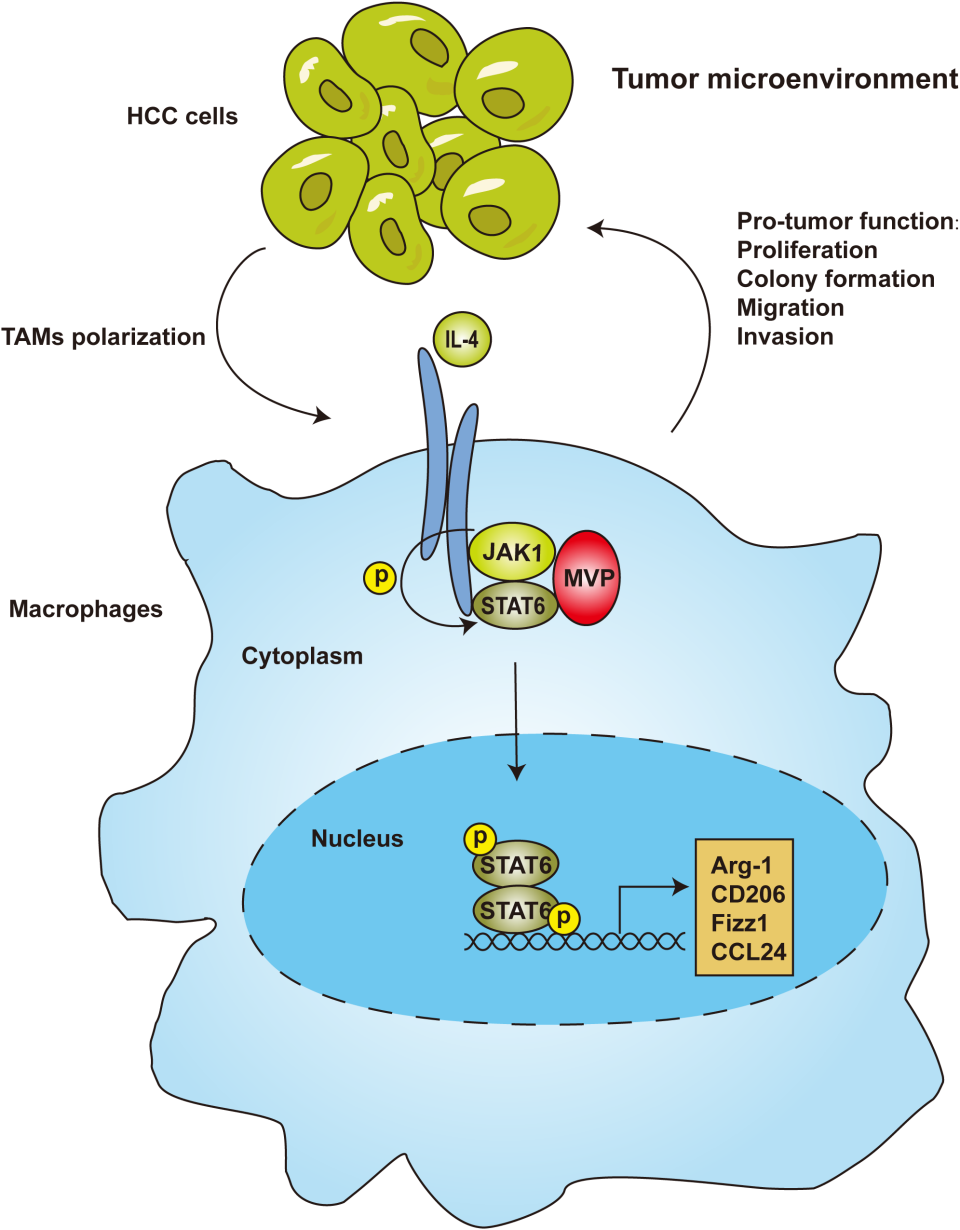
MVP-promoted M2-TAMs polarization and tumorigenesis in HCC. In the tumor microenvironment of hepatocellular carcinoma, JAK1 recruits MVP and STAT6, leading to ternary complex formation. Then, STAT6 is phosphorylated and translocated from the cytosol to the nucleus. As a result, STAT6 binds to the promoter of M2 genes, leading to M2 polarization and M2-TAMs infiltration.

We uncovered a novel regulatory pathway between macrophages and HCC development. We demonstrated that MVP increased HCC proliferation, metastasis and invasion by regulating the polarization of M2 macrophages. Mechanistically, MVP interacted with JAK1 and STAT6, and enhanced the recruitment of JAK1 to STAT6 in response to IL-4 treatment. As a result, STAT6 moves to the nucleus and binds to the promoter of M2 genes, ultimately initiating genetic transcription (**Figure 7**).

The 110-kDa MVP, also called lung resistance-related protein, is the dominant structural protein of the vault complex, and is involved in viral innate immunity, multidrug resistance, and cell signaling (18–20). However, the relationship between MVP and M2-TAMs has not yet been adequately studied in TME of HCC. Our previous studies showed that MVP contributes to HCC progression in animal models and MVP up-regulation correlates with several hallmarks of malignancy and associates with poor overall survival (21). Mechanistic studies showed that MVP associates with IFN regulatory factor 2 (IRF2) and disrupts IRF2/HDM2 complexes (21). This finding led us to investigate the role of MVP in the HCC tumor microenvironment. We found that MVP was positively correlated with the infiltration level of M2-like TAMs in HCC. The knockout of MVP in macrophages inhibited the phenotype of M2-like macrophages and reduced their promotional functions of proliferation, metastasis and invasion. Intriguingly, we found a significant co-localization between MVP and macrophage markers (**Figure 1**). Thus, MVP is not only high expressed in tumor cells but also in M2-like macrophages. The combined effect of elevated levels of MVP in HCC tumor and M2-like macrophages regulates HCC tumor microenvironment. Upon investigating the mechanisms behind this event, we found that MVP interacts with STAT6 and promotes the phosphorylation of STAT6 by facilitating the interaction between JAK1 and STAT6. Scaffold proteins operate as organizing hubs to enable high-fidelity signaling, fulfilling crucial roles in the regulation of cellular processes. Based on these results, we propose that MVP is a scaffold protein in tumor related signaling pathway. MVP organizes pro-tumoral cellular processes by bringing signaling molecules into interaction. It has not slipped our attention that the present findings suggest that MVP and the binding interface of MVP and STAT6 in particular might be interesting targets for future drug development. A landmark study shows that expression of MVP in insect cells that do not express VPARP and TEP1 gives rise to vault-like particles (31). MVP knockout disrupts the whole vault particle and eliminates or severely impairs its function (22). It will also be essential to determine whether MVP binds to STAT6 as a free protein or as part of the vault particle. When considering the next step, studies exploring these questions would be of great help in further clarifying the role of MVP in HCC.

Macrophages are highly plastic and change their phenotype and function according to the surrounding environment. Based on macrophage phenotype and functional characteristics, macrophages in TME can be classified into M1-TAMs that inhibit tumorigenesis and M2-TAMs that promote tumorigenesis (32). TAMs with M2-like macrophage phenotypes predominate in macrophage populations of TME (33). TAMs are abundant among tumor-infiltrating immune cells in most cancers (34). Significantly, infiltrating anti-inflammatory (M2-like) macrophages in the tumor microenvironment are associated with poor outcomes in HCC (35). In the HCC tumor microenvironment, M2-TAMs promote tumorigenesis by enhancing proliferation, invasion, metastasis, and angiogenesis; they also suppress anti-tumor immunity by inhibiting the functions of CD4^+^ T cells and CD8^+^ T cells (36). In this study, we found that *Mvp^-/-^* conditioned medium inhibit the pro-tumor function of M2 macrophages and the polarization function of Hepa1-6 cells (**Figure 3**). In our previous studies, we performed microarray analyses to identify MVP-regulated genes (18). A series of NF-κB-regulated genes and cytokines were up-regulated after transfection with pCMV-MVP (18). In light of our previous and current results, we propose a working model of the role of MVP in HCC (**Figure 7**). According to this model, in tumor cells, MVP associates with IRF2 and thereby promotes the secretion of pro-tumoral cytokines such as IL-4. Then, pro-tumoral cytokines bind to macrophage surface receptors and induce the interaction with MVP and STAT6, lead to the activation of M2-TAMs. Interestingly, there is an positive feedback loop: activated M2-TAMs also secrete various cytokines and chemokines to interact with tumor cells, lead to malignancy and poor overall survival of HCC patients (**Figure 7**).

Macrophages change their phenotype, controlled by chemokines and cytokine-mediated signaling pathways (37). For example, IFN-γ-mediated STAT1 signaling and LPS-mediated NF-κB signaling are classical pathways that promote M1 polarization (38, 39). A previous study showed MVP inhibited IFN-γ-mediated activation of STAT1 phosphorylation and nuclear translocation in lung cancer cells (40). However, no article systematically elucidated the relationship between MVP and macrophage polarization. Here, we demonstrated that MVP inhibits M1 macrophage polarization. Conversely, IL-4 stimulated the STAT6 signaling pathway that promotes M2 macrophage polarization. A recent study reported a relationship between MVP and M2 macrophage polarization in fracture repair but did not delve into the specific molecular mechanisms between MVP and IL4/STAT6 signaling pathway (41). In the present study, we revealed the molecular mechanism between MVP and IL4/STAT6 signaling pathway in detail. We found that MVP interacts with STAT6 and promotes the phosphorylation of STAT6 by facilitating the interaction between JAK1 and STAT6. STAT6 is translocated into nuclear and binds the promotor of M2-like associated specific genes. STAT6 is the major transcription factor responsible for the induction of M2 genes during M2 macrophage polarization. Several lines of evidence indicated that STAT6 transcriptional modulation regulates anti-tumor immunity, including phosphorylation, ubiquitination, and acetylation (29, 30, 42, 43). However, in this study, MVP only regulated the phosphorylation of STAT6, a specific role of MVP in STAT6 activation.

We previously reported that MVP promotes HCC by decreasing p53 activity (21). The present study found that MVP contributes to crosstalk between TAMs and tumor cells in the HCC tumor microenvironment. Mechanistically, MVP associates with STAT6, lead to STAT6 phosphorylation and nuclear localization. Although more studies are needed to understand the delicate regulatory mechanisms of MVP in TME-related HCC, our findings reveal a role for MVP in the regulation of TME-related HCC development.

## Data availability statement

The original contributions presented in the study are included in the article/**Supplementary Material**. Further inquiries can be directed to the corresponding authors.

## Ethics statement

The studies involving humans were approved by The institutional review board of Wuhan University. The studies were conducted in accordance with the local legislation and institutional requirements. The participants provided their written informed consent to participate in this study. The animal study was approved by The Institutional Review Board of Wuhan University. The study was conducted in accordance with the local legislation and institutional requirements.

## Author contributions

CY: Conceptualization, Data curation, Methodology, Writing – original draft. QZ: Methodology, Writing – review & editing. CM: Methodology, Writing – review & editing. CL: Methodology, Writing – review & editing. LN: Writing – review & editing. HC: Formal analysis, Writing – review & editing. QW: Formal analysis, Writing – review & editing. FW: Formal analysis, Writing – review & editing. HR: Visualization, Writing – review & editing. HY: Visualization, Writing – review & editing. KX: Writing – original draft. LZ: Formal analysis, Writing – review & editing. CZ: Visualization, Writing – review & editing. GL: Visualization, Writing – review & editing. ZL: Writing – review & editing. YZ: Conceptualization, Writing – review & editing. SL: Conceptualization, Writing – review & editing.
