# Supplementary material for: Major vault protein regulates tumor-associated macrophage polarization through interaction with signal transducer and activator of transcription 6
Source: Front Immunol. 2024 Jan 9;14:1289795. doi: 10.3389/fimmu.2023.1289795 (PMC10803552; doi:10.3389/fimmu.2023.1289795)
Supplement: Supplementary file 1 [file DataSheet_1.docx]

Supplementary Material

Supplementary Figures


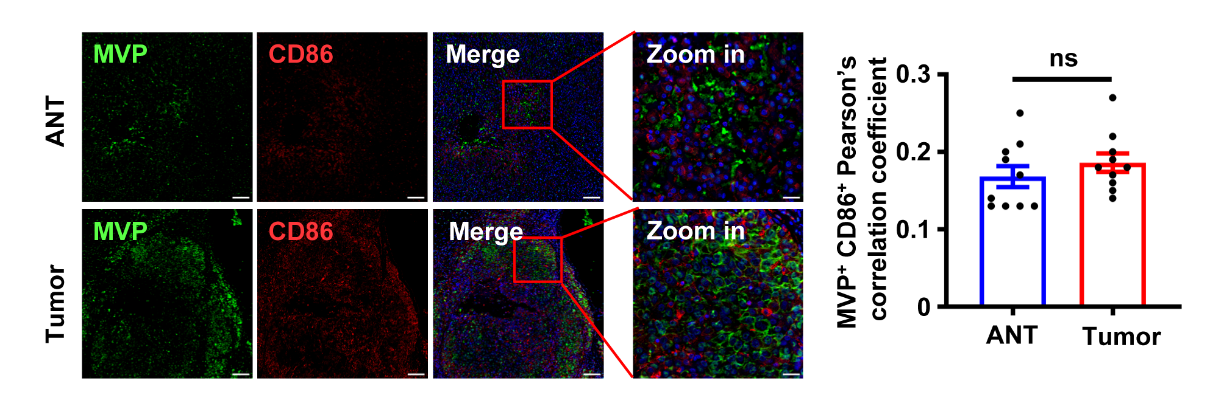


**Supplementary Figure 1.** **MVP negatively correlates with CD86^+^ TAMs in HCC samples. Related to Figure 1.** Immunofluorescence representative images and co-localization quantitative analysis of MVP (green) with CD86 (red) in adjacent nontumorous tissues (ANT) and HCC tumor tissues (n=10). Scale bar, 100 µm (MVP/CD86/Merge) and 20 µm (Zoom in). All levels of co-localization are indicated by Pearson’s correlation coefﬁcients calculated using Image J. Data are expressed as means ± SEM, two-tailed Student's t-test, (ns, no signiﬁcance).

**
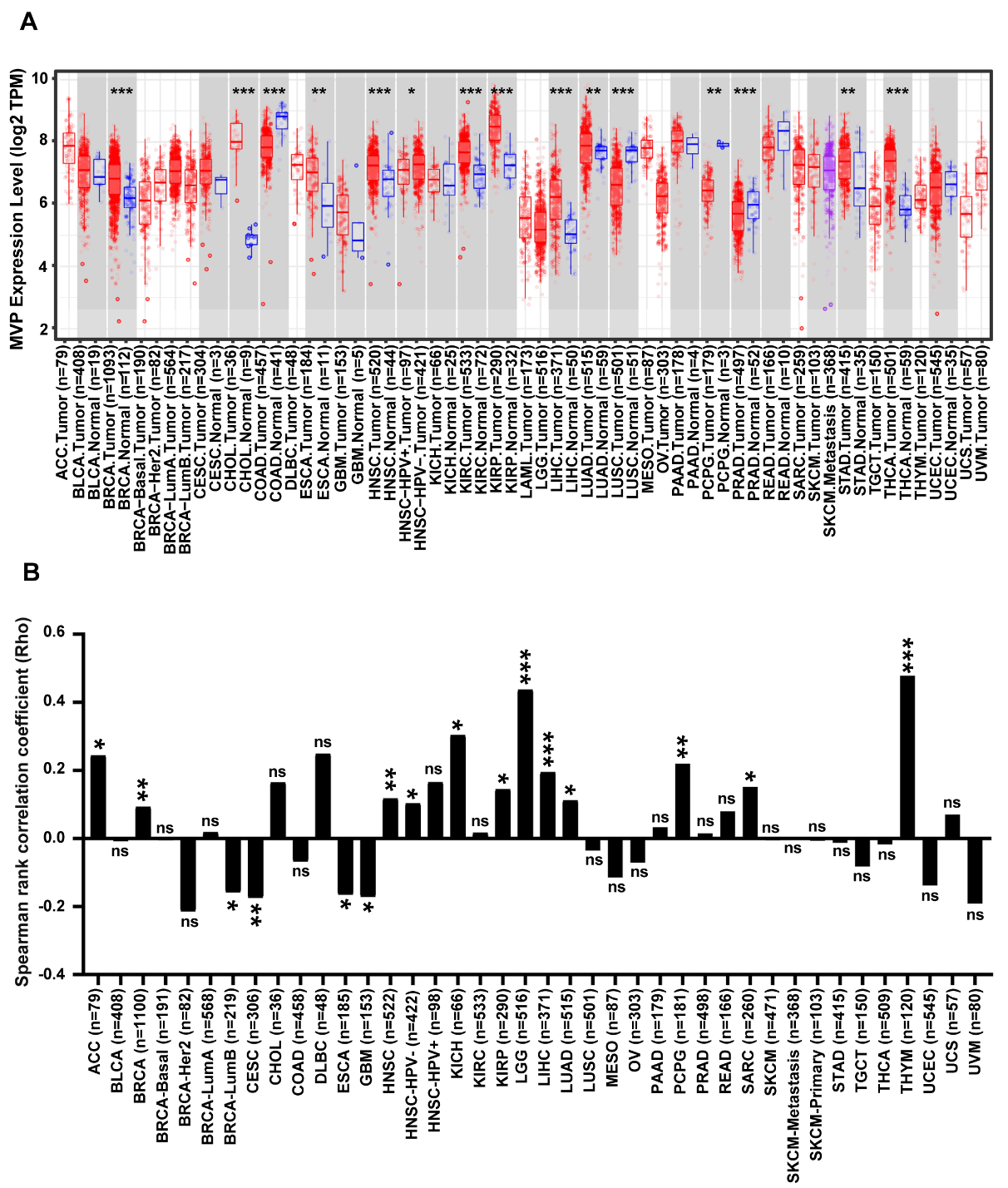
**

**Supplementary Figure 2. MVP positively correlated with CD68^+^/Arg-1^+^/CD206^+^ TAMs in hepatocellular carcinoma samples. Related to Figure 1.** (A) The box plots of MVP expression levels in human tumor types in TCGA detected by TIMER2.0. (B) The histogram of Rho between MVP expression and M2 macrophages infiltration across all TCGA tumors from the TIMER2.0 database. TPM: Transcripts Per Kilobase of exon model per Million mapped reads. Data are expressed as means ± SEM, two-tailed Student's t-test, (***P < 0.001, **P < 0.01, *P < 0.05, ns, no signiﬁcance).


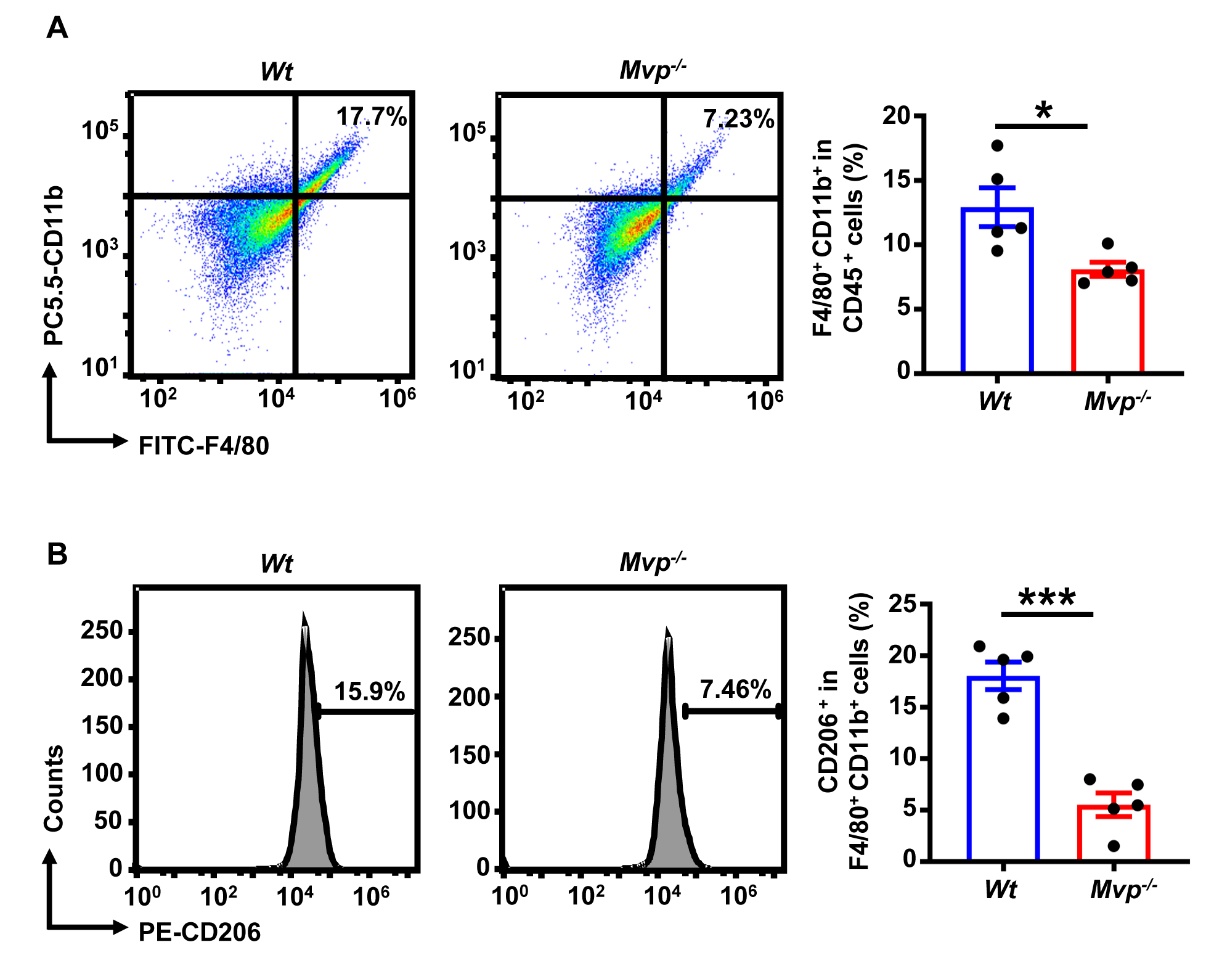


**Supplementary Figure 3.** **MVP knockout inhibits F4/80^+^ CD11b^+^ CD206^+^ TAMs *in vivo*.** **Related to Figure 2.** (A) FC analysis of TAMs infiltration in Hepa1-6 tumors in *Wt* mice and *Mvp^-/-^* mice (n = 5). The representative image of the percentage of F4/80^+^ CD11b^+^ cells in CD45^+^ cells (left panel). The percentage of positive cells out of total cells using Flow Jo (right panel). (B) FC analysis of M2-like TAMs infiltration in Hepa1-6 tumors in *Wt* mice and *Mvp^-/-^* mice (n = 5). The representative image of the percentage of CD206^+^ cells in F4/80^+^ CD11b^+^ cells (left panel). The percentage of positive cells out of total cells using Flow Jo (right panel).All data in the ﬁgure showed the means ± SEM, two-tailed Student's t-test, (***P < 0.001, *P < 0.05).


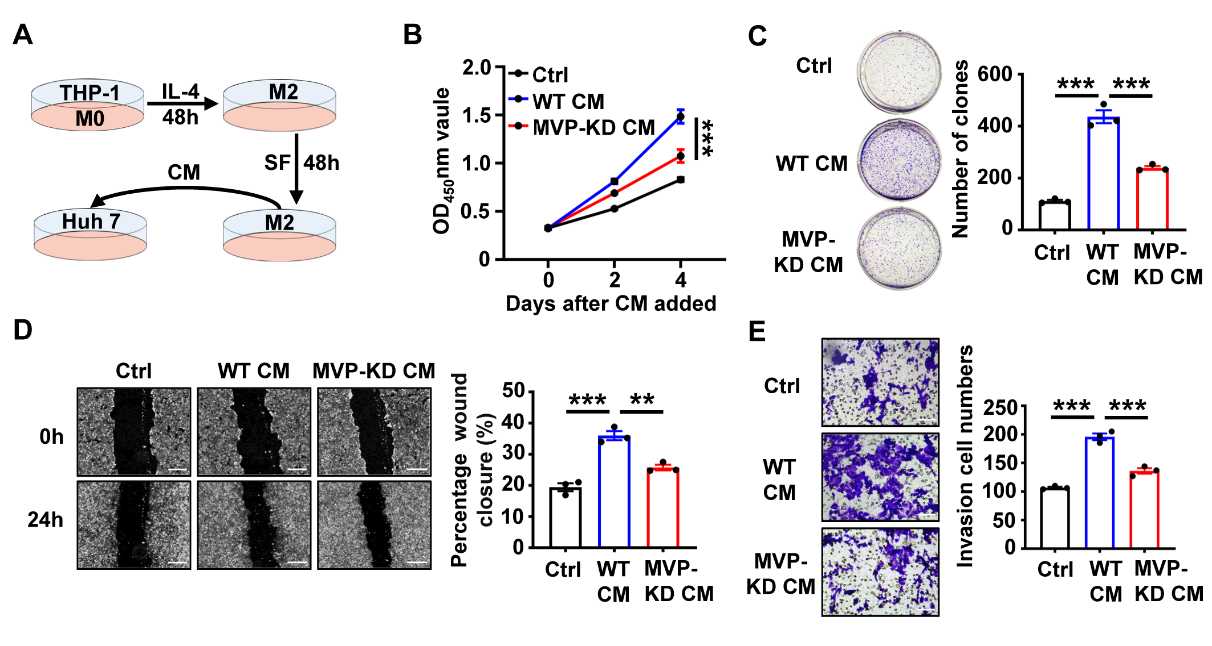


**Supplementary Figure 4.** **MVP modulates cell growth, motility under CM. Related to Figure 3.** (A) Model diagram of the THP-1 cells conditioned medium (CM) preparation and treatment. (B) Huh-7 cells were cultured in CM from WT or MVP knockdown (MVP-KD) THP-1 cells for 4 days, and cell vitality was measured via CCK-8 assays. (C) Representative image and quantitative analysis of clonal formation assay in Huh-7 cells cultured in CM for 21 days. The quantitative analysis was performed using Image J. (D and E) Huh-7 cells were cultured in CM for 24 hours (h) prior to wound healing assay (Scale bar, 50 µm) or transwell invasion assay (Scale bar, 50 µm). The quantitative analysis was performed using Image J. Data in this ﬁgure showed the means ± SEM, n = 3 per condition, one-way ANOVA, (***P < 0.001, **P < 0.01).


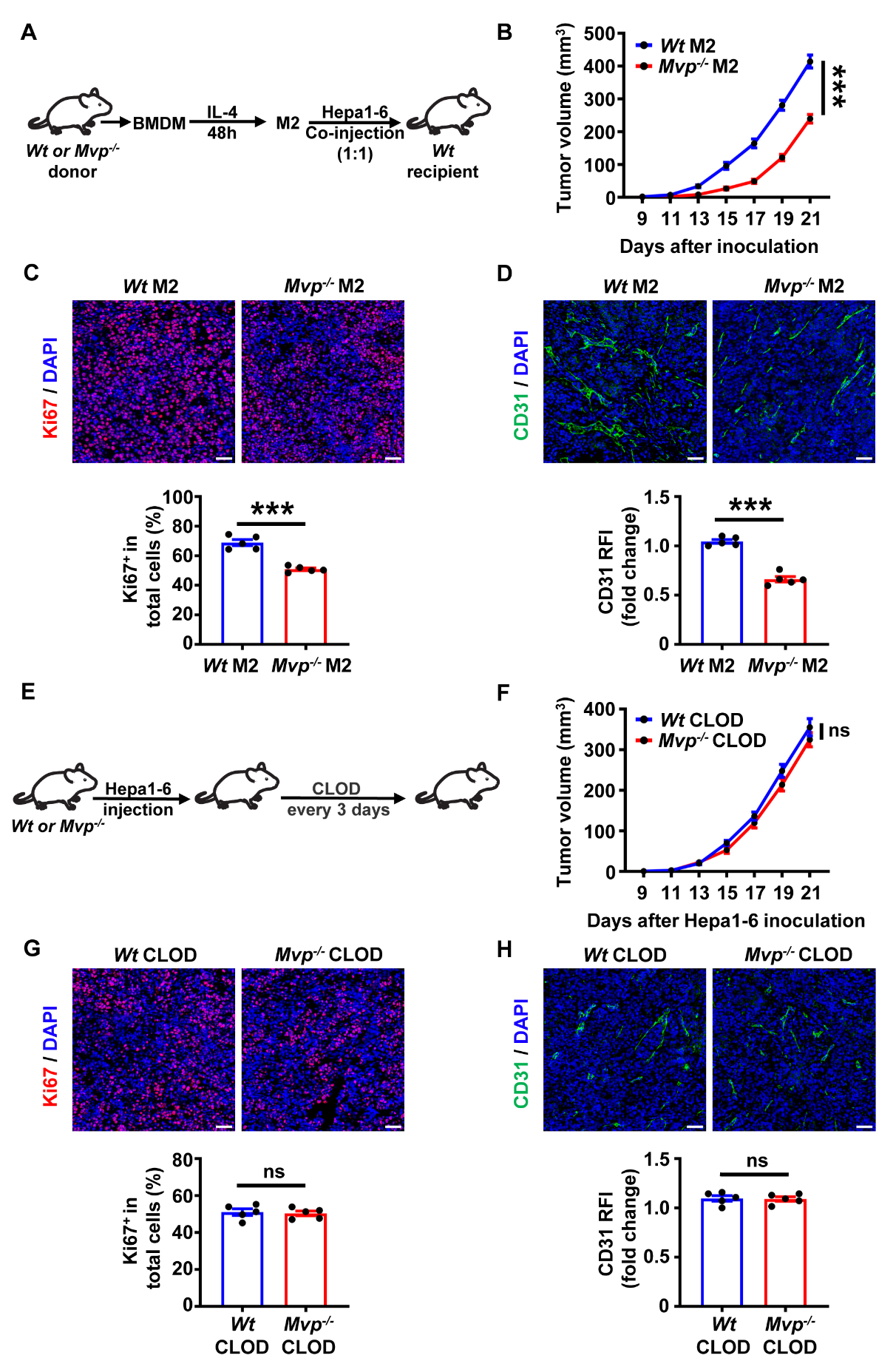


**Supplementary Figure 5. MVP modulates HCC tumorigenicity via macrophages in vivo. Related to Figure 3.** (A) Schematic protocol for co-injection tumor model. (B) Tumor growth curve. (C and D) Representative IF images and quantitative analysis of Ki67 (red) (C) and CD31 (green) (D) in tumor tissues (n=5). Scale bar, 50 µm. The percentage of positive Ki67 cells out of total cells and the relative fluorescence intensity (RFI) of CD31 were analyzed by Image J. (E) Schematic protocol for macrophage depletion tumor model. Clodronate liposomes (CLOD). (F) Tumor growth curve. (G and H) Representative IF images and quantitative analysis of Ki67 (red) (G) and CD31 (green) (H) in tumor tissues (n=5). Scale bar, 50 µm. The percentage of positive Ki67 cells out of total cells and the RFI of CD31 were analyzed by Image J. Data in (B and F) are presented as means ± SEM, two-way ANOVA. Data in (C, D, G, and H) are presented as means ± SEM, two-tailed Student's t-test. (***P < 0.001, ns, no signiﬁcance).


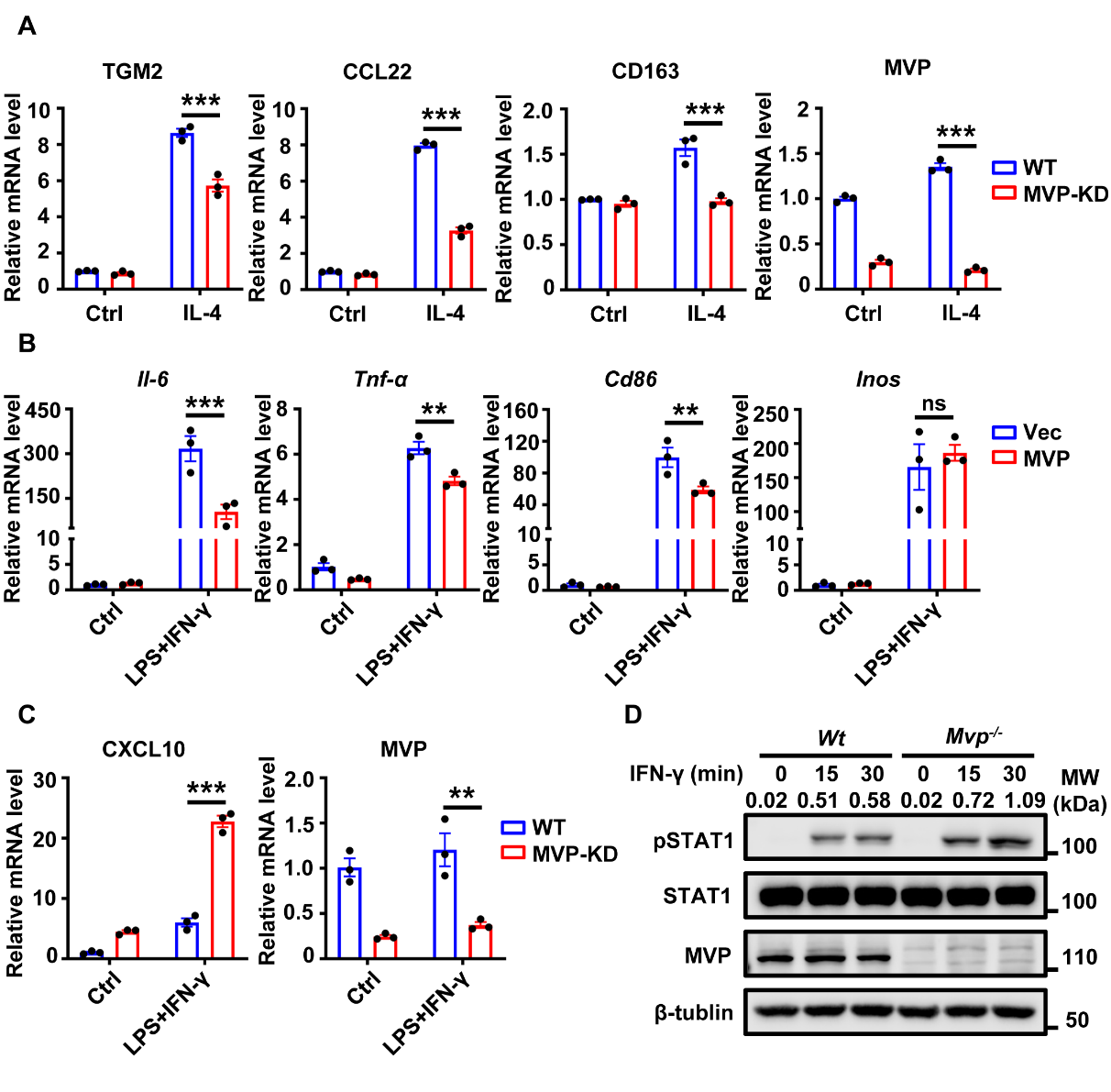


**Supplementary Figure 6. MVP regulates M2 macrophage polarization in response to IL-4 treatment. Related to Figure 4.** (A) qRT-PCR analysis of M2 gene expression in WT and MVP-KD THP-1 cells stimulated with medium or IL-4 for 24 h. (B) Flag-MVP plasmid or vector plasmid was transfected into Raw264.7 cells. At 24 h after transfection, cells were treated with or without LPS and IFN-γ for 24 h and then harvested for qRT-PCR analysis. (C) WT and MVP-KD THP-1 cells were stimulated with or without LPS and IFN-γ for 24 h and then harvested for qRT-PCR analysis of M2 gene expression. (D) *Wt* and *Mvp^-/-^* BMDMs were stimulated with or without LPS and IFN-γ at the indicated times before western blot analysis. All protein abundance analyses were performed using Image J. Data in this ﬁgure showed the means ± SEM, n = 3 per condition, two-way ANOVA. (***P < 0.001, **P < 0.01, ns, no signiﬁcance).


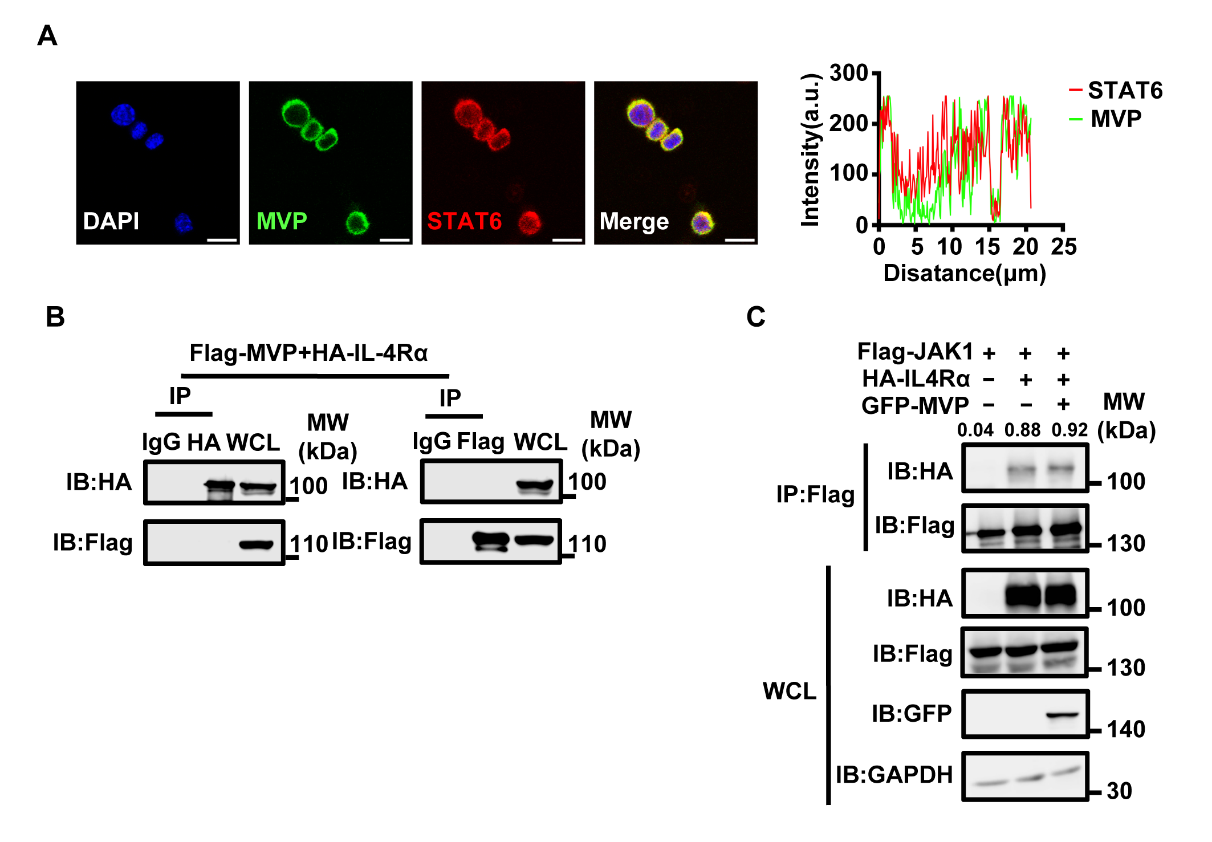


**Supplementary Figure 7. MVP interacts with STAT6 and JAK1. Related to Figure 5.** (A) Representative image of the co-localization MVP (green) and STAT6 (red) (left panel) in peritoneal macrophages (PMs). The quantitative analysis of the co-localization using Image J (right panel). Scale bar, 10 µm. (B) Flag-MVP, HA-IL-4Rα, and GFP-MVP were transfected into HEK293T cells for 36 h, and cells were collected for Co-IP and immunoblot analyses. (C) HEK293T cells were transfected with the indicated plasmids at 36 h post-transfection. The cells were collected, and performed Co-IP and immunoblot analyses. All protein abundance analyses were performed using Image J. All experiments were repeated at least three times with similar results.


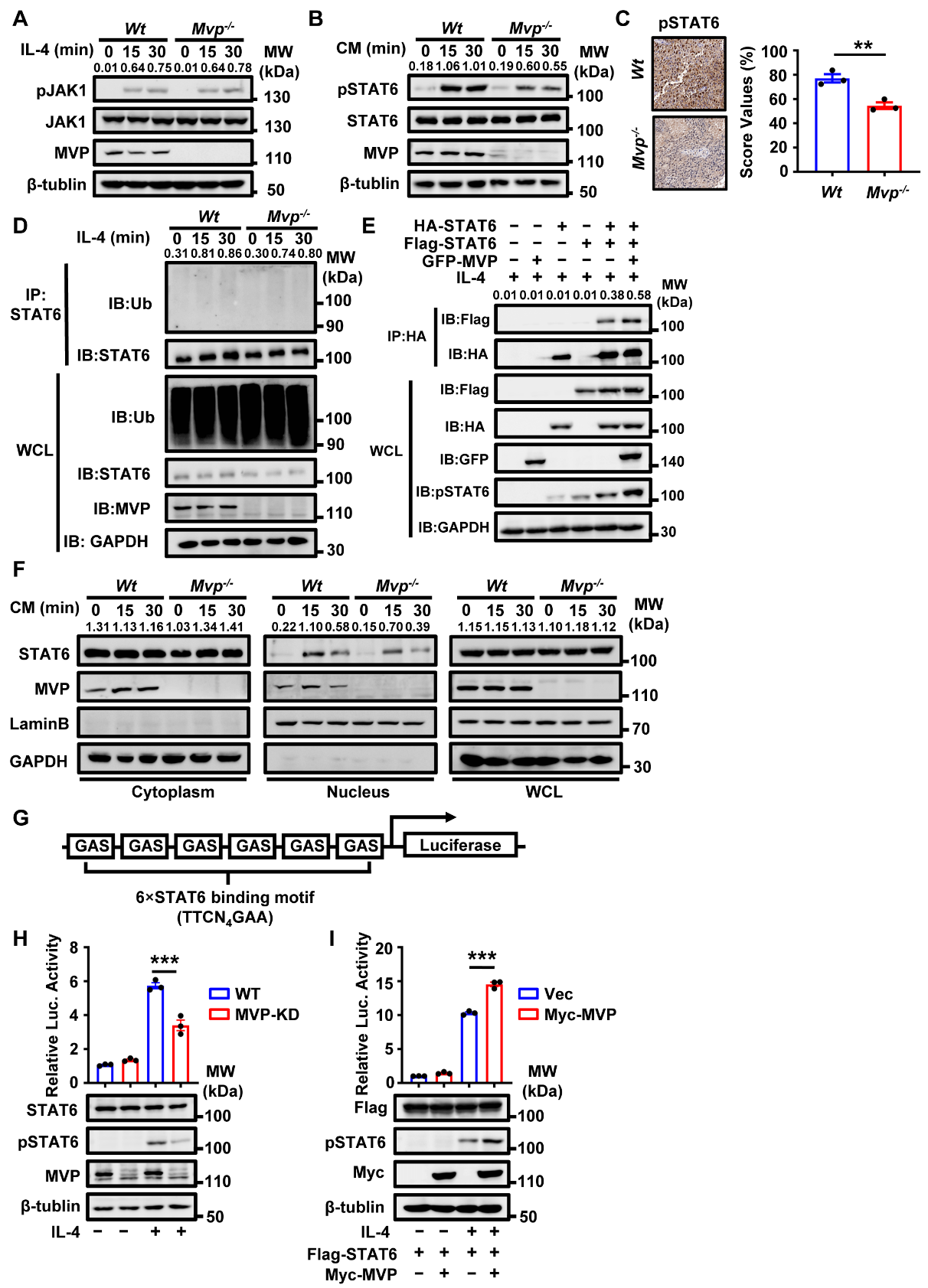


**Supplementary Figure 8. MVP enhances the phosphorylation and nuclear translocation of STAT6**. **Related to Figure 6.** (A) *Wt* and *Mvp^-/-^* BMDMs were treated with or without IL-4 at the indicated time and collected for immunoblot analyses. (B) *Wt* and *Mvp^-/-^* BMDMs were stimulated with or without Hepa1-6 cells CM at the indicated times before immunoblot analysis. (C) Representative image of IHC staining of pSTAT6 in Hepa1-6 cells induced HCC tumors from WT and MVPKO mice (n=3). Scale bar, 50 µm (left panel). (D) *Wt* and *Mvp^-/-^* BMDMs were treated with or without IL-4 at the indicated time. Co-IP and immunoblot analyses were performed with the indicated antibodies. (E) HEK293T cells were transfected with indicated plasmids for 36 h and treated with IL-4 for 1h. Co-IP and immunoblot analyses were performed with the indicated antibodies. (F) *Wt* and *Mvp^-/-^* BMDMs were stimulated with or without Hepa1-6 cells CM at the indicated time. The whole cell lysates (WCL), cytosolic and nuclear extracts were prepared and subjected to western blot analyses. Lamin B and GAPDH were used as nuclear and cytosolic fractions markers, respectively. (G) Schematic representation of STAT6 luciferase reporter plasmid (STAT6-luc) contains six consecutive STAT6 DNA-binding motifs (GAS). (H) WT and MVP-KD THP-1 cells were transfected with STAT6-luc for 36h and treated with or without IL-4 for 12 h before luciferase assays and western blot analysis. (I) Experiments were performed similar to those in (H), except indicated plasmid were transfected into HEK293T cells. All protein abundance analyses were performed using Image J. All experiments were repeated at least three times with similar results. Data in (C) are presented as means ± SEM, n = 3, two-tailed Student's t-test. Data in (H and I) are presented as means ± SEM, n = 3 per condition, two-way ANOVA. (***P < 0.001, **P < 0.01).

**Supplementary Tables**

**Supplementary Table 1.** The Spearman's Rho between MVP expression level and 22 types of immunocyte infiltration in 371 LIHC patients from TIMER2.0 database.

| **Infiltrating** **immunocyte** | **Spearman's Rho** | **P value** |
| --- | --- | --- |
| **Macrophage M0** | 0.17632031 | 0.00100572 |
| **Macrophage M1** | -0.1177155 | 0.02880541 |
| **Macrophage M2** | 0.19395275 | 0.00029018 |
| **T cell CD8^+^** | -2.96E-02 | 5.83E-01 |
| **T cell CD4^+^ naive** | -0.0345185 | 0.52281234 |
| **T cell CD4^+^ memory resting** | -0.0245319 | 0.64977562 |
| **T cell CD4^+^ memory activated** | 0.0637118 | 0.23788142 |
| **T cell regulatory (Tregs)** | 0.11909167 | 0.02697503 |
| **B cell plasma** | -0.0005299 | 0.99217526 |
| **B cell naive** | -0.0940205 | 0.08118006 |
| **B cell memory** | 3.02E-02 | 5.76E-01 |
| **Neutrophil** | 0.13845947 | 0.01002835 |
| **Myeloid dendritic cell resting** | 0.10720806 | 0.04661158 |
| **Myeloid dendritic cell activated** | -0.0254601 | 0.6374547 |
| **Monocyte** | -0.1739444 | 0.00117901 |
| **NK cell resting** | -0.1204586 | 0.02525684 |
| **NK cell activated** | -0.057662 | 0.28550901 |
| **T cell gamma delta** | -9.27E-02 | 8.56E-02 |
| **T cell follicular helper** | -0.075676 | 0.16075727 |
| **Eosinophil** | 0.12464427 | 0.02056917 |
| **Mast cell resting** | 0.03492541 | 0.51792084 |
| **Mast cell activated** | -0.1109305 | 0.03946322 |

**Supplementary Table 2.** Clinical data of 10 HCC patients used in this study.

| **No.** | **Sex** | **Age**  **(years)** | **Tumor size**  **(cm)** | | **TNM stage** | **Tumor differentiation** | |
| --- | --- | --- | --- | --- | --- | --- | --- |
| **1** | M | 42 | | 11×8×7 | T3NxMx | | Moderately |
| **2** | M | 57 | | 2.5×2.1×2 | T2NxMx | | Moderately |
| **3** | M | 65 | | 2.2×2.2×1.5 | T1bNxMx | | Well |
| **4** | F | 78 | | 2.7×2.2×1 | T1NxMx | | Moderately |
| **5** | M | 69 | | 10.5×10.2×8.9 | T3N0Mx | | Moderately |
| **6** | M | 37 | | 15×8×7 | T1bN0M0 | | Moderately |
| **7** | F | 59 | | 3.8×3×2.5 | T1bN0M0 | | Moderately-poorly |
| **8** | M | 43 | | 1×1×1.2 | T1aN0M0 | | Moderately |
| **9** | M | 16 | | 22×20×13.5 | T1bN0M0 | | Well |
| **10** | F | 67 | | 3×3×2  1.5×1.5×0.6 | T2N0M0 | | Moderately |

*TNM: Tumor Node Metastasis*

**Supplementary Table 3.** The P value of the differential expression between tumor and adjacent nontumorous tissues for MVP in TCGA cancers from TIMER2.0 database.

| **Tumor tissues** | **ANT** | **P value** |
| --- | --- | --- |
| BLCA.Tumor (n=408) | BLCA.Normal (n=19) | 0.67635496 |
| BRCA.Tumor (n=1093) | BRCA.Normal (n=112) | 1.98E-16 |
| CESC.Tumor (n=304) | CESC.Normal (n=3) | 0.162878783 |
| CHOL.Tumor (n=36) | CHOL.Normal (n=9) | 2.26E-09 |
| COAD.Tumor (n=457) | COAD.Normal (n=41) | 4.15E-16 |
| ESCA.Tumor (n=184) | ESCA.Normal (n=11) | 0.004273766 |
| GBM.Tumor (n=153) | GBM.Normal (n=5) | 0.249248204 |
| HNSC-HPV+.Tumor (n=97) | HNSC-HPV-.Tumor (n=421) | 0.016196059 |
| HNSC.Tumor (n=520) | HNSC.Normal (n=44) | 2.32E-06 |
| KICH.Tumor (n=66) | KICH.Normal (n=25) | 0.596791904 |
| KIRC.Tumor (n=533) | KIRC.Normal (n=72) | 9.64E-20 |
| KIRP.Tumor (n=290) | KIRP.Normal (n=32) | 1.80E-14 |
| LIHC.Tumor (n=371) | LIHC.Normal (n=50) | 8.39E-14 |
| LUAD.Tumor (n=515) | LUAD.Normal (n=59) | 0.007692384 |
| LUSC.Tumor (n=501) | LUSC.Normal (n=51) | 3.73E-18 |
| PAAD.Tumor (n=178) | PAAD.Normal (n=4) | 0.523353132 |
| PCPG.Tumor (n=179) | PCPG.Normal (n=3) | 0.003407601 |
| PRAD.Tumor (n=497) | PRAD.Normal (n=52) | 0.000954487 |
| READ.Tumor (n=166) | READ.Normal (n=10) | 0.081600908 |
| SKCM.Tumor (n=103) | SKCM.Metastasis (n=368) | 0.272626811 |
| STAD.Tumor (n=415) | STAD.Normal (n=35) | 0.00795136 |
| THCA.Tumor (n=501) | THCA.Normal (n=59) | 5.80E-22 |
| UCEC.Tumor (n=545) | UCEC.Normal (n=35) | 0.167971218 |

*ANT: adjacent* *nontumorous tissues*

**Supplementary Table 4.** The Spearman's Rho between MVP expression level and M2 macrophages infiltration level in TCGA cancers from TIMER2.0 database.

| **Cancer** | **Spearman's Rho** | **P value** |
| --- | --- | --- |
| **ACC (n=79)** | 0.24331942 | 0.038049006 |
| **BLCA (n=408)** | -0.007204198 | 0.890452155 |
| **BRCA (n=1100)** | 0.092523038 | 0.003504373 |
| **BRCA-Basal (n=191)** | 0.003450152 | 0.96396166 |
| **BRCA-Her2 (n=82)** | -0.213575803 | 0.071642913 |
| **BRCA-LumA (n=568)** | 0.017782111 | 0.686671377 |
| **BRCA-LumB (n=219)** | -0.158131848 | 0.028478373 |
| **CESC (n=306)** | -0.174197792 | 0.003632978 |
| **CHOL (n=36)** | 0.164332235 | 0.345512499 |
| **COAD (n=458)** | -0.066889436 | 0.268976902 |
| **DLBC (n=48)** | 0.24799973 | 0.117958453 |
| **ESCA (n=185)** | -0.16470731 | 0.027140025 |
| **GBM (n=153)** | -0.171215768 | 0.045450157 |
| **HNSC (n=522)** | 0.117444753 | 0.009121841 |
| **HNSC-HPV- (n=422)** | 0.10276034 | 0.039954798 |
| **HNSC-HPV+ (n=98)** | 0.16515321 | 0.121943222 |
| **KICH (n=66)** | 0.303084226 | 0.014121598 |
| **KIRC (n=533)** | 0.016265073 | 0.727614247 |
| **KIRP (n=290)** | 0.143992983 | 0.020683673 |
| **LGG (n=516)** | 0.436315997 | 1.23E-23 |
| **LIHC (n=371)** | 0.193952752 | 0.000290184 |
| **LUAD (n=515)** | 0.11131383 | 0.013399019 |
| **LUSC (n=501)** | -0.035393887 | 0.440572 |
| **MESO (n=87)** | -0.114758409 | 0.295646639 |
| **OV (n=303)** | -0.071178753 | 0.263161437 |
| **PAAD (n=179)** | 0.032717023 | 0.670977839 |
| **PCPG (n=181)** | 0.219363368 | 0.004395572 |
| **PRAD (n=498)** | 0.015059261 | 0.759419615 |
| **READ (n=166)** | 0.079266053 | 0.457698536 |
| **SARC (n=260)** | 0.151648394 | 0.017769575 |
| **SKCM (n=471)** | 0.004613902 | 0.921642977 |
| **SKCM-Metastasis (n=368)** | -0.004859941 | 0.92740012 |
| **SKCM-Primary (n=103)** | 0.003344519 | 0.973386087 |
| **STAD (n=415)** | -0.013246644 | 0.797144745 |
| **TGCT (n=150)** | -0.082490053 | 0.320566337 |
| **THCA (n=509)** | -0.018032286 | 0.691104247 |
| **THYM (n=120)** | 0.478015512 | 6.57E-08 |
| **UCEC (n=545)** | -0.13851259 | 0.198094873 |
| **UCS (n=57)** | 0.070263742 | 0.617111051 |
| **UVM (n=80)** | -0.191200247 | 0.095765374 |

**Supplementary Table 5.** Primer Sequence used in this study.

| **Target Gene** | **Purpose** | **Primer** **Sequence (5' to 3')** |
| --- | --- | --- |
| **m-*Gapdh*-F** | RT-PCR | CCTGCACCACCAACTGCTTAG |
| **m-*Gapdh*-R** | RT-PCR | GTGGATGCAGGGATGATGTTC |
| **m-*Arg-1*-F** | RT-PCR | TTTTTCCAGCAGACCAGCTT |
| **m-*Arg-1*-R** | RT-PCR | AGAGATTATCGGAGCGCCTT |
| **m-*Cd206*-F** | RT-PCR | CAGGTGTGGGCTCAGGTAGT |
| **m-*Cd206*-R** | RT-PCR | TGGCATGTCCTGGAATGAT |
| **m-*Fizz1*-F** | RT-PCR | GGGATGACTGCTACTGGGTG |
| **m-*Fizz1*-R** | RT-PCR | TCAACGAGTAAGCACAGGCA |
| **m-*Ccl24*-F** | RT-PCR | ATTCTGTGACCATCCCCTCAT |
| **m-*Ccl24*-R** | RT-PCR | TGTATGTGCCTCTGAACCCAC |
| **m-*Il-6*-F** | RT-PCR | TACCACTTCACAAGTCGGAGGC |
| **m-*Il-6*-R** | RT-PCR | CTGCAAGTGCATCATCGTTGTTC |
| **m-*Tnf-α*-F** | RT-PCR | GTTCTATGGCCCAGACCCTCAC |
| **m-*Tnf-α*-R** | RT-PCR | GGCACCACTAGTTGGTTGTCTTTG |
| **m-*Inos*-F** | RT-PCR | GAGACAGGGAAGTCTGAAGCAC |
| **m-*Inos*-R** | RT-PCR | CCAGCAGTAGTTGCTCCTCTTC |
| **m-*Cd86*-F** | RT-PCR | ACGTATTGGAAGGAGATTACAGCT |
| **m-*Cd86*-R** | RT-PCR | TCTGTCAGCGTTACTATCCCGC |
| **m-*Mvp*-F** | RT-PCR | GGACCAGATGGCAAGAACCA |
| **m-*Mvp*-R** | RT-PCR | CTGCTCTGACAGCACGTACA |
| **h-GAPDH-F** | RT-PCR | GTCTCCTCTGACTTCAACAGCG |
| **h-GAPDH-R** | RT-PCR | ACCACCCTGTTGCTGTAGCCAA |
| **h-TGM2-F** | RT-PCR | TGTGGCACCAAGTACCTGCTCA |
| **h-TGM2-R** | RT-PCR | GCACCTTGATGAGGTTGGACTC |
| **h-CCL22-F** | RT-PCR | GAGCATGGATCGCCTACAG |
| **h-CCL22-R** | RT-PCR | CAGACGGTAACGGACGTAATC |
| **h-CD163-F** | RT-PCR | TTCACTGCACTGGGACTGAG |
| **h-CD163-R** | RT-PCR | AGGACAGTGTTTGGGACTGG |
| **h-CXCL10-F** | RT-PCR | CCTTATCTTTCTGACTCTAAGTGGC |
| **h-CXCL10-R** | RT-PCR | ACGTGGACAAAATTGGCTTG |
| **h-MVP-F** | RT-PCR | GGCAGGACAATGAGAGGGTACT |
| **h-MVP-R** | RT-PCR | CCGAACTTGCCCTGTGACAT |

*m: mouse; h: human*
